# Long term dietary *Moringa oleifera* leaf extract to Florida red tilapia *Oreochromis* sp improves performance immunity maturation and reproduction in saltwater

**DOI:** 10.1038/s41598-025-06881-2

**Published:** 2025-06-23

**Authors:** Ghada R. Sallam, Mohamed M. Abdel-Rahim, Ayman M. Lotfy, Walied M. Fayed, Akram Ismael Shehata, Mohammed F. El Basuini, Rasha I. Elwan, Mohamed A. Al-absawey, Ashraf I. G. Elhetawy

**Affiliations:** 1 Aquaculture Division, National Institute of Oceanography and Fisheries, NIOF, Cairo, Egypt; 2 Animal and Fish Production Department, Faculty of Agriculture, Alexandria University, Saba-Basha, P.O. Box 21531, Alexandria, Egypt; 3 Animal Production Department, Faculty of Agriculture, Tanta University, Tanta, 31527 Egypt; 4 Faculty of Desert Agriculture, King Salman International University, South Sinai, Sinai Egypt

**Keywords:** Tilapia, *Moringa oleifera*, Salinity, Immunostimulatory, Sex hormones, Reproductive function, Biological techniques, Ecology, Immunology, Physiology, Zoology

## Abstract

High salinity impairs offspring production in Florida red tilapia (FRT) *Oreochromis* sp. A total of 624 FRT broodstock (1:1 ratio of ♂: ♀) were divided into 16 groups, with 4 males and 4 females housed separately at two salinity levels (18 ppt and 32 ppt). Fish were fed four different levels of *Moringa oleifera* leaf extract (MOLE) supplementation (0, 5, 10, and 15 g MOLE kg^−1^ diet) for two months. Following the initial feeding period, males and females receiving the same MOLE level under the same salinity conditions were transferred to 24 spawning concrete tanks. The experiment consisted of eight groups, each containing 3♂ and 6♀, with triplicate setups (four groups at 18 ppt and four groups at 32 ppt). Fish were fed at 1% of their body weight for four months. The results revealed significant (p *< 0.05*) improvements in water quality (lower ammonium and nitrite), growth parameters, feed conversion ratio, carcass protein content, digestive enzymes, liver enzymes, cortisol level, innate immunity, antioxidants, testosterone and progesterone hormones, and reproductive function (♂ and ♀) with MOLE-fed broodstock in both salinities. MOLE at 10–15 g/kg can improve FRT performance, welfare, fertility (♀), and reproduction under high salinity conditions (32 ppt).

## Introduction

Tilapia possess morphological and biological characteristics that make their cultivation disseminate rapidly throughout the globe, across diverse aquaculture systems and conditions in more than 135 countries and regions^1,2^. Tilapia is featured by its big size, rapid growth, fertile reproductive traits, palatability, relatively low production cost, and reaching maturity within two to three months of hatching^3–5^. Tilapia farming has developed to occupy the second position among cultivated fish species globally and tops all species in Egypt^6^. In 2020, multinational tilapia production represented 12.5% of the global cultivated species, with Egypt leading the list of producers after China and Indonesia^7,8^. Tilapias are low-nutrient fish and their omnivorous food conventions make them relatively cheap to feed, as they can accept higher levels of dietary fiber and carbohydrates than most other farmed fish^9,10^. Dietary protein requirements for tilapia decline with age and size, from 30 to 56% for fry, and 30–40% for juveniles to 28–30% protein for larger tilapia^9,11^. Furthermore, the optimum dietary lipid was found to be 5.2% for fry weighing up to 2.5 g and 4.4% for fish weighing up to 7.5 g, while it should be between 8 and 12% for tilapia weighing up to 25 g, before dropping to 6–8% for larger fish^9,11–13^. Therefore, practicing balanced feed (requiring 10 essential amino acids, n-6 linoleic fatty acid, and n-3 linolenic fatty acid) is crucial for the success of tilapia farming to ensure high productivity and rapid growth^9,13^. In this regard, many natural additives have been applied to tilapia feeds as growth promoters and immunostimulants, such as *Zingiber officinale*^14–16^ *Thymus vulgaris*^14,16,17^ *Cinnamomum camphora*^15,18^ *Echinacea purpurea*^15,19^ and *Moringa oleifera*^16,20^ but not to study the positive impacts on performance and reproductive traits under salinity challenges.

Tilapia can tolerate a wide range of environmental conditions, including salinity, dissolved oxygen, temperature, pH, and ammonia levels^9,21^. Most tilapias (except *Oreochromis niloticus*) have a high tolerance to saltwater, although salinity tolerance varies between species and profoundly affects some physiological functions such as reproduction^9,21,22^. Among the different tilapia hybrids currently being farmed, the red tilapia hybrids are of utmost preference for marketable aquaculture because of their biological characteristics (shape and appearance) and their capacity to grow in a wide range of salinities up to full-strength marine water^21,23,24^. Besides salinity tolerance, red tilapia has a combination of valuable features not found in most other tilapias, such as rapid growth, adaptability to most farming methods, lack of a black membrane in the body cavity, and elevated marketing prices compared to other black tilapia^23,25^. The genetic distinctions in red tilapia are generally thought to have arisen from the hybridization of blue tilapia (*O. aureus*) and Nile tilapia with the reddish-orange Mozambique tilapia (*Oreochromis mossambicus*)^26,27^. The Florida red tilapia (FRT) *Oreochromis mosambicus* × *Oreochromis hornorum*, or red-gold strain, was developed in the 1970 s as a result of mating a male Mozambique tilapia (*Oreochromis mossambicus*) with a female Wami tilapia (*Oreochromis hornorum*)^28,29^. Although FRT can thrive in waters with salinity levels up to 36.2%, high salinity poses a major challenge to propagation and seed production^21,29^.

Salinity is a crucial factor affecting sperm viability in aquatic animals, including tilapia, while changes in salinity can entail raised energy expense to keep body fluid balance, particularly to regulate sodium and chloride ions^30,31^. Furthermore, steroid hormones play a key role in regulating the immune system, and androgens can suppress or stimulate the level of immunity and thus may boost or deteriorate the health and growth of tilapia^32^. The gene network associated with innate immune responses is influenced by androgen levels, as demonstrated by decreased levels of red blood cells and lymphocytes in Nile tilapia treated with 17α-methyldihydrotestosterone^33^ while the disruptive impact on antioxidant enzyme capacity and gene transcription was seen after feeding tilapia fry^32,34^. In the same vein, environmental stressors, including salinity, can affect aquatic organisms, causing undesirable short- or long-term consequences, such as cytogenetic toxicity, embryonic malformations, impaired fish fertility, and delayed hatching^35,36^. Therefore, enhancing fish reproduction requires comprehending the crucial aspects impacting this paramount physiological function^37^. Therefore, emphasizing the key role of feed quality and composition in mitigating the adverse effects of higher salinity on FRT propagation and spotlighting its impact through salinity forbearance during broodstock maturation and spawning is a point worthy of research and discussion. Prior investigations have emphasized the significance of energy control for triumphant fish reproduction and highlighted the necessity for specialized feeding during broodstock preparation to fulfill both qualitative and quantitative energy needs^38–40^. Because of the considerable energy expenditure in egg formation and sperm production, maternal nutrition plays a critical part in triumphant osmoregulation and propagation^38^. This opens the portico for phyto derivatives, specifically *M. oleifera* (miracle tree) extracts, as a conceivable solution to improve feed efficiency, alleviate the inhibitory effect of elevated salinity, and thus boost reproductive function.

*M. oleifera* prospers in tropical and subtropical areas and can adapt to a wide range of different environmental conditions^41,42^. Roughly all parts (root, seeds, bark, seed oil, gum, leaves, fruit, and flowers) of this tree have been employed for special beneficial uses and have shown advantageous impacts on health, nourishment, wastewater, and the environment^41^. *M. oleifera* exhibits distinctive advantageous therapeutic characteristics such as anti-inflammatory, antibacterial, and antifungal properties^43^ antioxidant^44^ immunostimulant^45^ and hepatoprotective properties^46^. Moreover, *M. oleifera* grain cake is commonly employed as a naturalistic biofloc in water remedy implementations^47^ while root extracts have been shown to impede the growth of pathogens such as *Klebsiella pneumonia* and *Listeria monocytogenes*^48^. Furthermore, *M. oleifera* is rich in macro- and micronutrients, including vitamins, minerals, crude fat (106 g/kg) and crude protein (260 g/kg) in dry matter, polyunsaturated fatty acids, essential amino acids, flavanol glycosides and phytochemicals such as carotenoids, phenolic acids, and alkaloids^41,49,50^. Several studies have indicated the praising influences of *M. oleifera* supplements in improving the performance and well-being of many aquatic species, including Nile tilapia^51–53^ freshwater prawn, *Macrobrachium rosenbergii*^44^ *Cirrhinus mrigala*^54^ and Bocourti’s catfish, *Pangasius bocourti*^55^. Also, some studies have shown that *M. oleifera* leaves are good for the quality of sperm in male trout, *Oncorhynchus mykiss*^50^ and for the levels of estradiol and testosterone in Nile tilapia^56,57^. However, no previous studies have been launched to explore the potential impact of *M. oleifera* supplementation in supporting FRT mating in saline water and alleviating the adverse effect of high salinity on broodstock fertility and reproductive performance, particularly ♀.

Considering the incorporated influences of reproduction and high salinity above 18 ppt in impairing offspring production, this work seeks to assess the effect of *M. oleifera* leaf ethanol extract (MOLE) supplementation on the reproductive and fertility function of FRT broodstock in both moderate and high salinity environments. This work aims to evaluate the potential impact of MOLE supplementation on culture water, growth performance, feed efficiency, carcass quality, digestion enzymes, immune parameters, antioxidant capacity, and reproductive hormones (testosterone and progesterone) with a focus on the major obstacle of poor reproduction (maturity, fertility, spawning, egg and sperm viability, and hatchability) of FRT in saline water. This will open up prospects for employing these desirable fish to develop desert aquaculture in new areas using saline groundwater and conserving freshwater for human, animal, and plant uses, thus overcoming the issue of freshwater scarcity.

## Materials and methods

### Fish facility and experimental location

A total of 624 apparently healthy FRT broodstocks (312♂ mean weight = 123.04 ± 0.15 g/fish and mean length = 15.4 ± 0.22 cm; and 312♀ mean weight = 96.20 ± 1.19 g/fish and mean length = 14.1 ± 0.17 cm) were selected from the broodstock foundation raised in outdoor concrete tanks (3 × 10 × 1.5 m each, with a stocking density of 208 fish/tank) at a salinity of 36 ppt in the Marine Fish Farming Unit, Kilo 21 (GAFRD), and transferred to a private fish farm in Wadi Mariout, Alexandria, Egypt. Prior to commencing the experiment, the fish were sexed manually through the examination of their genital papillae. They were then placed in separate rearing tanks equipped with an air blower for two weeks to acclimate to the experimental environment. Both ♂ and ♀ were adapted to the experimental salinities of 18 and 32 ppt^21,22^ during this time, consuming a basal diet (BD) until they reached satiation in a natural light cycle (12 h light and 12 h dark). The Fish Nutrition Lab (Department of Fish and Animal Production, Faculty of Agriculture Saba-Basha Alexandria University), in collaboration with the Fish Rearing Lab NIOF, Alexandria, Egypt, completed the experiment in two phases: pre-spawning and spawning.

### Diet preparation and experimental layout

#### *M. oleifera* leaf extract (MOLE) preparation

Pure and dried *M. oleifera* leaves were acquired from the Imtenan store, a local market located in Alexandria Governorate, Egypt. The leaves were pulverized into a fine powder and filtered through a fine-mesh screen. Subsequently, the substances were combined with a solution containing 80% ethanol in a 1:1 proportion^58,59^ and allowed to be immersed for a duration of 48 h. The solution was passed through filter paper to isolate any leftover residue from the leaves, and the resulting solution was condensed using a rotary evaporator at a temperature of 40 °C. The final product of *M. oleifera* leaf extract was preserved at a temperature of 4 °C until it was ready for use. The *M. oleifera* leaf ethanol extract (MOLE) was prepared using the method outlined by Abidin, et al.^60^.

#### Experimental diet preparation

A private fish feed facility (Aquafeed International for Food Industries, Motobes, Kafr El-Shaikh, Egypt) formulated the experimental basal diet (BD). The BD was supplemented with four levels of MOLE: 0.0, 5.0, 10.0, and 15.0 g/kg of diet^53,61^. These levels were referred to as D0, D5, D10, and D15 diets, respectively. The proximate analysis of the basal diet supplemented with different doses of MOLE is represented in Table 1. The MOLE was dissolved in distilled water to obtain a concentration of 10% w/v and then applied as a spray to the diets of all of the experimental groups. The diet was then dehydrated in a forced-air oven, maintaining a temperature range of 45–50 °C^62^. The selected levels of MOLE extract were chosen based on the research conducted by Emam et al.^63^. The fish feed was stored at a temperature of 4 °C until it was used. The fish were provided with the experimental diets for a duration of 6 months, consisting of a pre-spawning period of 60 days, followed by a spawning period of 120 days.

#### Experimental layout

The study was divided into two phases: pre-spawning (phase A) and spawning (phase B). The FRT received MOLE supplementation during both phases, under a moderate salinity (MS) of 18 ppt and a high salinity (HS) of 32 ppt. Four levels (0, 5, 10, and 15 g/kg) of MOLE supplementation were included in the basal diet (BD) to formulate 4 diets (D0, D5, D10, and D15) used to feed eight fish groups (4 groups at 18 ppt and 4 groups at 32 ppt) as follows:

(MSD0) where fish fed BD without MOLE at 18 ppt salinity;

(MSD5) where fish fed BD containing 5 g kg^−1^ MOLE at 18 ppt salinity;

(MSD10) where fish fed BD containing 10 g kg^−1^ MOLE at 18 ppt salinity;

(MSD15) where fish fed BD containing 15 g kg^−1^ MOLE at 18 ppt salinity;

(HSD0) where fish fed BD without MOLE at 32 ppt salinity;

(HSD5) where fish fed BD containing 5 g kg^−1^ MOLE at 32 ppt salinity;

(HSD10) where fish fed BD containing 10 g kg^−1^ MOLE at 32 ppt salinity;

(HSD15) where fish fed BD containing 15 g kg^−1^ MOLE at 32 ppt salinity.

#### Pre-spawning experiment (phase A)

This phase lasted 60 days and included housing ♂ and ♀ in separate tanks. During this phase, male and female FRT were randomly assigned to 48 outdoor circular fiberglass tanks (1.25 m^3^ each) at a density of 13 fish per tank. Under each salinity (18 and 32 ppt), 8 groups (4♂ and 4♀) were placed in triplicate (24 tanks) with each group fed with one level of MOLE, as shown in Table 2. The fish received a diet equal to 3% of their body weight three times a day, seven days a week, during this stage. At the end of the pre-spawning phase, we gathered and counted all the fish in each group. A total of 9 ♂ and 18 ♀ of comparable weight were randomly selected to enter the spawning phase with the same MOLE dose and salinity level. No mortality had been recorded among fish within this period.

#### Spawning experiment (phase B)

This phase lasted for 120 days using greenhouse-occupied concrete tanks that were cleaned and prepared to receive the broodstock for mating, with salinity adjusted to meet the experimental conditions. To start this phase, 18 ♀ and 9 ♂ (6♀ and 3♂for each replicate)^64,65^ were chosen at random from the variant treatment in different group tanks and transferred to the spawning tanks with the same treatment (salinity and diet) for the mating process, with a male-to-female ratio of 1:2. A total of 24 spawning concrete tanks (2 × 1 × 1 m each), representing 8 groups (each 3♂+ 6♀) in triplicate (4 groups at 18 ppt and 4 groups at 32 ppt), with each 3 ♂ and 6 ♀ fed the same MOLE dose under the same salinity level at phase (A) were placed in one tank (Table 2 displays the experimental design). Fish received experimental diets at 1% of their body weight three times a day, seven days a week, for 120 days. Every 15 days, the fish were weighed, and female brooders checked for fry and/or eggs. Each batch of spawn was taken out of the brooders, numbered, weighed, and preserved separately to ensure the quality of the offspring produced by each group. For the whole experiment, the tanks were consistently aerated, and the fish were maintained on a natural light cycle of 12 h of light and 12 h of darkness.

#### Water quality management

During the acclimation and experimental phases, the tanks were cleaned every day, and solid waste was eliminated. The daily water exchange was around 30% using a flow-through water exchange system, which had the same salinity and temperature as the treatment water and was constantly aerated. The water temperature (°C), pH, salinity (mS/cm converted to ppt), and dissolved oxygen (O_2_) were measured daily on-site using a multiparameter portable photometer at specific times (10:00–10:30 AM). Ammonium (NH_4_+), nitrate (NO_3_^−^) and nitrite (NO_2_^−^) were measured weekly during the experimental period, according to APAH^66^.

### Evaluated parameters after a six-month spawning experiment

#### Performance variables and biometric indicators

Following the six-month trial, all broodstock (♂ and ♀) in each tank were gathered, allocated numbers, and weighed. Multiple parameters, including growth performance, utilization of feed, survival rate, carcass chemical analyses, and organ indices, were systematically recorded and assessed using the subsequent equations^22,56^:

$${\text{Weight gain }}\left( {{\text{WG}}} \right),{\text{ g}}/{\text{fish }} = {\text{ final body weight }}\left( {{\text{FBW}}} \right){\text{ }}{-}{\text{ initial body weight }}\left( {{\text{IBW}}} \right)$$

$${\text{Average daily gain }}\left( {{\text{ADG}}} \right),{\text{ g}}/{\text{fish}}/{\text{day }} = {\text{ WG}}/{\text{period }}\left( {{\text{days}}} \right)$$

$${\text{Specific growth rate }}\left( {{\text{SGR}}} \right),{\text{ }}\% /{\text{fish}}/{\text{day }} = {\text{ }}\left[ {{\text{Ln FBW}} - {\text{Ln IBW}}/{\text{period}}} \right]{\text{ }} \times {\text{ 1}}00$$

$${\text{Survival rate}},{\text{ }}\% {\text{ }} = {\text{ Final number}}/{\text{Initial number }}*{\text{ 1}}00$$

$${\text{Condition factor }}\left( {{\text{CF}}} \right){\text{ }} = {\text{ body weight }}\left( {\text{g}} \right)/{\text{body length }}\left( {{\text{cm}}} \right)^{{\text{3}}} \times {\text{ 1}}00$$

$${\text{Feed intake }}\left( {{\text{FI}}} \right),{\text{ g}}/{\text{fish}}/{\text{experimental period }}\left( {{\text{days}}} \right){\text{ }} = {\text{ Eaten feed }}\left( {\text{g}} \right)/{\text{fish number}}$$

$${\text{Feed conversion ratio }}\left( {{\text{FCR}}} \right){\text{ }} = {\text{ FI }}\left( {\text{g}} \right){\text{ }}/{\text{ WG}}$$

$${\text{Hepatosomatic index }}\left( {{\text{HSI}}} \right){\text{ }} = {\text{ Liver weight }}\left( {\text{g}} \right)/{\text{body weight}}\left( {\text{g}} \right)$$

$${\text{Viscerosomatic index }}\left( {{\text{VSI}}} \right){\text{ }} = {\text{ Viscera weight }}\left( {\text{g}} \right){\text{ }}/{\text{ body weight }}\left( {\text{g}} \right)$$

$${\text{Testosomatic index }}\left( {{\text{TSI}}} \right){\text{ }} = {\text{ Tests weight }}\left( {\text{g}} \right){\text{ }}/{\text{ body weight }}\left( {\text{g}} \right)$$

$${\text{Gonadosomatic index }}\left( {{\text{GSI}}} \right){\text{ }} = {\text{ Gonad weight }}\left( {\text{g}} \right)/{\text{body weight }}\left( {\text{g}} \right)$$

#### Whole-body proximate analysis

Prior to starting the reproduction study, a random selection of 5 ♂ and 5 ♀ was made and stored at −20 °C for later whole-body chemical analysis. Following the experiment, 6 fish (3♂ and 3♀) from each treatment group were chosen and kept at a temperature of −20 °C while awaiting analysis. Triplicate analysis of the moisture, lipid, protein, and ash content of both diets and fish samples was conducted following the approach described in AOAC^67^.

#### Digestive enzymes

By the end of the experiment, three fish were chosen at random from each tank, with a total of nine fish per treatment, and subjected to digestive enzyme examination. The fish were rendered anesthetized with 0.50 mg clove oil per liter, and their intestines were carefully surgically removed. As described by Makled et al.^68^. the intestinal contents were gathered, thoroughly mixed with a cold sucrose solution (0.25 M) in glass test tubes using a tissue homogenizer coated with Teflon, and then subjected to centrifugation at 5000 × g for 30 min at 4 °C. Following extraction, the supernatant was gathered and kept at 4 °C for enzymatic testing within 24 h Suzer et al.^69^. The specific activity was quantified as U mg^−1^ of intestinal content. Measurement of protease activity was conducted with bovine serum albumin as the reference standard. The activity of amylase was assessed using starch as the substrate. The activity of lipase was quantified using β-naphthyl caprylate as the working substrate. Each unit of lipase activity was quantified as the release of 1 mg of β-naphthol every minute. The determination of intestinal amylase, protease, and lipase was conducted following the method described by Zamani, et al.^70^.

#### Sperm and egg quality and fecundity

After the pre-spawning experiment, fresh semen was obtained from five mature ♂ in each tank to evaluate the quality standards of the sperm. A comprehensive assessment of sperm quality was conducted by extracting seminal fluid from the remaining five adult ♂ in each tank simultaneously. Under a light microscope at 400× magnification, sperm motility was subjectively assessed using a five-category classification scheme (0; 0–25; 25–50; 50–75; or > 75%) based on the Boussit^71^. technique. Sperm concentration was quantified using a Neubauer counting chamber under a microscope, and sperm viability was assessed by the Eosin-Nigrosin staining technique^72^. Concurrently, the reproductive performance parameters were assessed in five mature ♀ chosen at random from each tank, while the other five fish were not included in the study. After death, precision incisions were made in the ovaries with a sharp knife to get samples of eggs.

The measurements included the weights, numbers, and diameters of eggs per female of the species. Quantification of egg count was conducted per gram of eggs and subsequently linked to either the ovarian weight or the body weight of the fish. Following that, the absolute fecundity (AF) and relative fecundity (RF) were computed using the equations proposed by Bhujel^73^ equations: AF = Absolute fecundity refers to the number of eggs that a female can release during a single reproductive season, and RF = the number of eggs that a female can release per unit weight (g) or unit length (mm) during a reproductive season.

#### Blood samples, hematological analysis, and serum biochemical assays

Fish in all tanks fasted for 24 h after feeding the tested diets. Every experimental treatment was sampled once, with three fish per tank for the measurement of hematological indices and three fish per tank for the analysis of plasma content. To anesthetize the fish, clove oil was administered at a concentration of 0.50 mg/L of water. Blood samples were then obtained by puncturing the caudal vein. Blood samples were extracted and divided into two tubes: one containing heparin for hematological examination and another without anticoagulants for biochemical assessment. The hematological tests were conducted using the Model 2000 Evolution, a semi-automatic analyzer produced by EMEG.

Red blood cell (RBC) count was quantified with a Bürker counting chamber together with Hayem solution. The red blood cell (RBC) count and total white blood cell (WBC) count were determined according to the standardized methods outlined by Hrubec, et al.^74^. Hct was determined using heparinized microhematocrit capillary tubes and a microhematocrit centrifuge set at 10,000× g for 5 min. The hematocrit values were obtained within a 30-minute period after the occurrence of bleeding. Hemoglobin levels (Hb, gm/dL) were determined by the cyanhemoglobin technique^75^ at a 540 nm emission wavelength. For this aim, a hemoglobin reagent kit from Zeist Chem Diagnostics, CA, USA, was employed. Using the technique outlined by Fischbach and Dunning^76^. the RBC indices, including mean corpuscular volume (MCV), mean corpuscular hemoglobin (MCH), and mean corpuscular hemoglobin concentration (MCHC), were calculated. The packed cell volume (PCV) was determined by using the standard method described by Stoskopf^77^. White blood cells (WBCs) and hemoglobin (Hb) were detected within 6 h after blood sample collection, and the process of distinguishing different WBC types was carried out.

The biochemical characteristics of the serum were examined using a biochemical kit obtained from Bio-Diagnostic Co. in Cairo, Egypt. The measured biochemical markers include total protein (TP), albumin (ALB), globulin (GLO), cholesterol (CHO), alanine aminotransferase (ALT), aspartate aminotransferase (AST), urea, and creatinine. The biuret procedure, as described by Doumas, et al.^78^ was used to estimate the total plasma protein concentration in grams per deciliter (g dL^−1^). ALB concentration (g dL^−1^) was determined by the bromocresol green reagent method as described by Reinhold^79^. By deducting the ALB concentration from the TP concentration, the concentration of GLO (g dL^−1^) was determined. The cortisol (COR) concentration (pg mL^−1^) was measured following the method defined by Foster and Dunn^80^. The serum CHO was measured using the method described by Trinder^81^. The kidney function markers, urea and uric acid, were evaluated according to the approach outlined by Whitehead, et al.^82^. Measurements of serum AST and ALT levels were conducted using the procedure described by Bergmeyer, et al.^83^.

#### Sex hormones

Progesterone and testosterone levels in the blood serum were measured using commercially available enzyme-linked immunosorbent assay (ELISA) test kits. The kits utilized were BC-1113 for progesterone and BC-1115 for testosterone, both manufactured by BioCheck, Inc. The methodology followed was as described by Tietz^84^.

#### Antioxidants and immunity indices

In order to assess the activity of antioxidant enzymes, the livers of three fish per replication were carefully removed and placed on the surface of ice. After that, they were placed in a refrigerator set at −20 °C. In accordance with the manufacturer’s guidelines, the liver samples were homogenized in a cold solution of 0.86% NaCl using a VEVOR, FSH-2 A homogenizer. The homogenate was centrifuged for 10 min at a temperature of 4 °C with a rotational speed of 12,000 rpm. A microplate was used to collect the supernatant and quantify enzyme activity precisely and reliably. A calorimetric technique was employed to identify superoxide dismutase (SOD), catalase (CAT), and glutathione peroxidase (GPx) at wavelengths of 550 nm, 280 nm, and 412 nm, respectively, using the methods described by Shahin et al.^85^; Aebi^86^ and Paglia and Valentine^87^. The measurement of malondialdehyde (MDA) levels was conducted using the Uchiyama and Mihara^88^ method. The technique described by Kim and Austin^89^. is the method for quantifying the enzymatic activity of serum lysozyme (U mg^−1^). Estimation of serum complement 3 (C3) concentration was conducted using ELISA kits (Fish complement 3 kits) purchased from MyBioSource. Specific ELISA kits were used to test total immunoglobulin M (IgM) concentration^90^.

#### Reproductive performance

On a daily basis, the female FRTs in each tank were examined for fertile eggs. Brooding ♀ were gently netted, and the eggs were properly collected from their oral cavity. An enumeration, weighing, and computation of the mean egg weight were performed on the collected eggs. The remaining eggs were labeled and promptly frozen for the purpose of chemical examination.

The following reproductive parameters were determined:

1. Time to first spawning (days) = duration from the date of stocking until the first spawning.
2. Total egg count per tank over the period of the study.
3. Average absolute fecundity = egg count per tank/the total number of ♀.
4. Average number of eggs per spawning = total egg count per tank/the number of spawning.
5. Total number of spawning per tank.
6. Average number of spawning per female = total number of spawning/total number of ♀.
7. Inter-spawning intervals (ISI; days) = The time interval between successive spawnings of repeating spawning fish (♀) only^91^.

#### Egg hatchability

From the females’ jaws, the eggs of the second or third spawn were taken and transferred into hatching jars containing the same culture water. Values for hatchability percentage, “days to hatch,” and yolk-sac absorption times were documented. Following the completion of yolk sac absorption, 10 swim-up fry were collected by nets, and their length was measured in mm.

### Statistical analysis

A statistical analysis was performed to evaluate the impact of the innovative method, including the addition of MOLE, on enhancing FRT embryo production under challenging conditions. The results are displayed as the mean ± standard errors of the means (SEM). Using SPSS software V26, data from the MOLE-complemented groups (MSD5, MSD10, MSD15, HSD5, HSD10, and HSD15) underwent a two-way ANOVA with post hoc analysis and a Duncan test to evaluate differences among the treatments under research. All differences exhibited statistical significance with a P-value less than 0.05. The statistical process tracked the procedure summarized by Assaad, et al.^92^. The data were assessed for normality using the Shapiro-Wilk’s test.

## Results

### Water quality assessment in spawning tanks

Table 3 illustrates the potential effect of dietary MOLE on the water quality parameters in FRT tanks across different salinity levels. Regarding the physicochemical parameters of water, no significant differences (*p* < 0.05) were recorded between treatments with respect to temperature, while O_2_ and pH fluctuated significantly due to the dietary addition of MOLE. Both O_2_ and pH fluctuated within the acceptable range for fish growth^93^ in all groups.

Furthermore, MOLE supplementation had a significant impact (*p <* 0.05) in reducing nitrogen by-products (NH_4_+, NO_2_-, and NO_3_-) in all tested groups compared to the control group at both salinities. While salinity alone did not affect NH4+, NO2- and NO3-, with their values ​​peaking in both MSD0 and HSD0 groups under both salinities, the effect of salinity-MOLE interaction was dose-dependent. The lowest NH4 + levels were recorded in the MSD15 and HSD10 groups, and the lowest NO2- values ​​were observed in the HSD5, and HSD10 groups, while the MSD15, HSD5, and HSD15 groups maintained lower NO3- levels than all groups.

### Growth indices and feed utilization

Dietary inclusion of MOLE in the FRT diet significantly affected growth performance (Table 4) and feed efficacy (Table 5) under both salinities. The growth parameters of fish fed the diets supplemented with MOLE were significantly improved compared to the control group. The improved FBW, WG, ADG, SGR, and CF were evident in both sexes across different salinities, especially in fish that were fed D10 and D15 (10 g and 15 g MOLE/kg diet). Moreover, the MOLE diet resulted in enhanced feed efficiency, as indicated by lower FCR values than with the control diet. The interaction effect of MOLE and salinity was dose-dependent and altered FRT performance in both sexes (*p* < *0.05*). Increasing MOLE dose resulted in higher FBW, WG, and ADG and lower FCR in both sexes under both salinities. MOLE administration at 15 g kg/diet at 32 ppt maintained the best FBW and SGR (♂ and ♀) and WG and ADG (♂) as recorded under 18 ppt while enhancing the FCR in both sexes to approach the value recorded at 18 ppt. Salinity alone had significant impacts on FBW, WG, ADG SGR, and FCR in female broodstocks. That influence, however, did not extend to ♂.

### Carcass chemical composition and biometric indices assessment

Table 6 delivers the effect of dietary MOLE and salinity on carcass composition of FRT. The inclusion of MOLE considerably (*p <* 0.05) varied the whole carcass composition of both ♂ and ♀, as demonstrated by increased protein and ash contents and lowered lipid value compared to the control group. Notably, these changes occurred as a result of a dose-dependent interaction between salinity and MOLE. Compared to the control group, the interaction between MOLE doses and MS (18 ppt) did not affect moisture content (♀) while increased in ♂. Both ♂ and ♀ fed three levels of MOLE across varying salinities showed higher protein and ash contents and lower lipids than those fed a control diet. Both sexes (♂ and ♀) showed relatively similar responses to the measured parameters at both salinities. Salinity alone played a significant role in changing the body chemical composition in both sexes, as documented by the differences between the MSD0 and HSD0 groups. Fish that were given diets supplemented with MOLE showed reductions in values of inner organ indicators (HSI and VSI) and improvements in gonad formation (TSI and GSI) as shown in Table 7. The interaction between MOLE and salinity had a remarkable influence on the development of the inner organ in FRT, with the highest TSI recorded in the MSD15 group, while the HSD15, MSD15, MSD10, and MSD5 groups shared the highest GSI.

### Digestive enzyme activity in FRT fed different levels of MOLE supplements

**Fig. 1:**
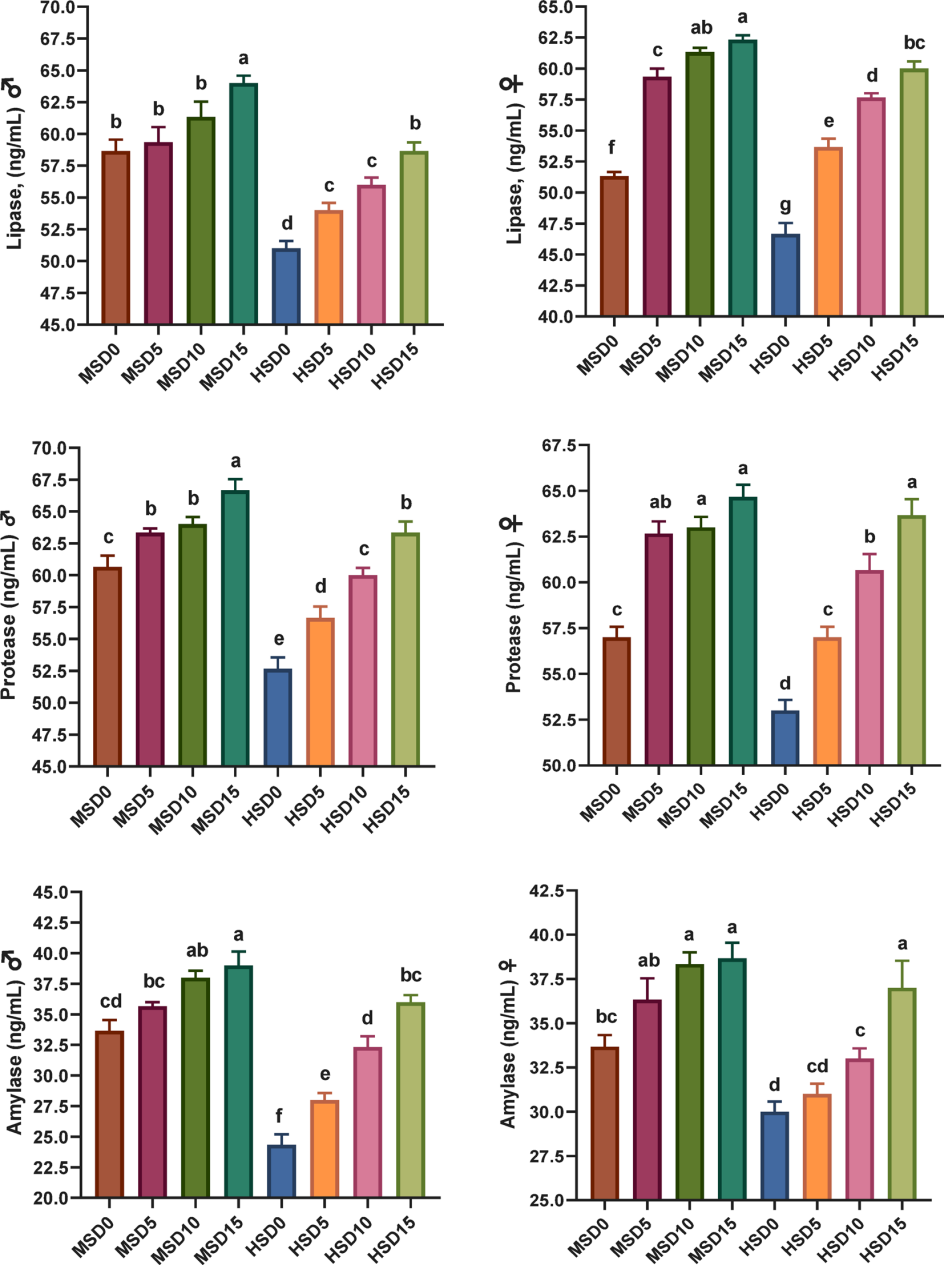
Digestive enzyme activity in Florida red tilapia (♂ and ♀) fed Moringa oleifera leaf ethanol extract (MOLE) for 60 days pre-spawning and 120 days spawning period, across different salinities. MSD0 = fish fed D0 diet @ 18ppt salinity; MSD5 = fish fed D5 diet @ 18ppt salinity; MSD10 = fish fed D10 diet @ 18ppt salinity; MSD15 = fish fed D15 diet @ 18ppt salinity; HSD0 = fish fed D0 diet @ 32 ppt salinity; HSD5 = fish fed D5 diet @ 32 ppt salinity; HSD10 = fish fed D10 diet @ 32 ppt salinity; HSD15 = fish fed D15 diet @ 32 ppt salinity; D0 = basal diet without MOLE-supplementation (control); D5 = MOLE-supplemented diet with 5 g/kg; D10 = MOLE-supplemented diet with 10 g/kg; D15 = MOLE-supplemented diet with 15 g/kg.

Figure 1 illustrates the enzymatic activity in FRT (♂ and ♀) when fed control and MOLE-supplemented diets at different salinities (18 ppt and 32 ppt). Salinity and MOLE interaction had a significant effect on all three digestive enzymes, which was dose-dependent. In ♂, compared to control, the interaction between salinity and MOLE dose significantly improved the three enzymes at 32 ppt, while at 18 ppt amylase in the MSD5 group and lipase in the MSD5 and MSD10 groups had no effect. In ♀, except for amylase in the MSD5 and HSD5 groups, the interaction between salinity and MOLE dose significantly enhanced the three enzymes across both salinities, compared to the control. The influence of salinity alone was significant as the lowest values ​​of the three enzymes were recorded in fish at 32 ppt salinity. All three enzymes demonstrated significant diet-salinity interactions, with fish in the groups MSD15 and HSD15 (15 g/kg MOLE at18 ppt and 32 ppt) showing the highest activity levels in both sexes. The activity of all digestive enzymes recorded higher values in fish (♂ and ♀) reared at 18 ppt compared to those reared at 32 ppt indicating a significant effect of salinity on both sexes.

*Haemato-biochemical indices.* Tables 8 and 9 illustrate the effects of diet and salinity on CBC, serum CHO, COR, liver enzymes (ALT and AST), and kidney function markers (urea and uric acid) in FRT broodstocks. Both factors remarkably influenced most parameters (*p* < 0.05), frequently interactively (MOLE × salinity). For both sexes, MOLE administration increased red blood cells (RBCs), white blood cells (WBCs), hemoglobin (Hb), and hematocrit (Hct) while lessening CHO, AST, and ALT. COR showed a dependent interaction response between salinity, MOLE dose, and sex, showing a significant decline in ♀ fed MOLE at both salinities, while ♂ showed a significant decrease in COR levels in the MSD5, HSD10 and HSD15 groups only compared to the control group. Except for ♀ fed 5 a g/kg MOLE diet at 32 ppt (group HSD5), all fish fed MOLE diets showed significant reductions in urea and uric acid contents compared to the control group at both salinities. In general, salinity lowered RBCs, Hb, Hct, and WBC, and increased CHO, ALT, AST, urea, uric acid, and COR in both sexes. Statistically significant effects of salinity and MOLE interaction on blood parameters of FRT were observed in both ♂ and ♀ (*p* < 0.05). Nevertheless, this impact was MOLE dose-dependent with some factors such as urea, uric acid, and COR.

### Innate immune parameters and antioxidant activity

**Fig. 2:**
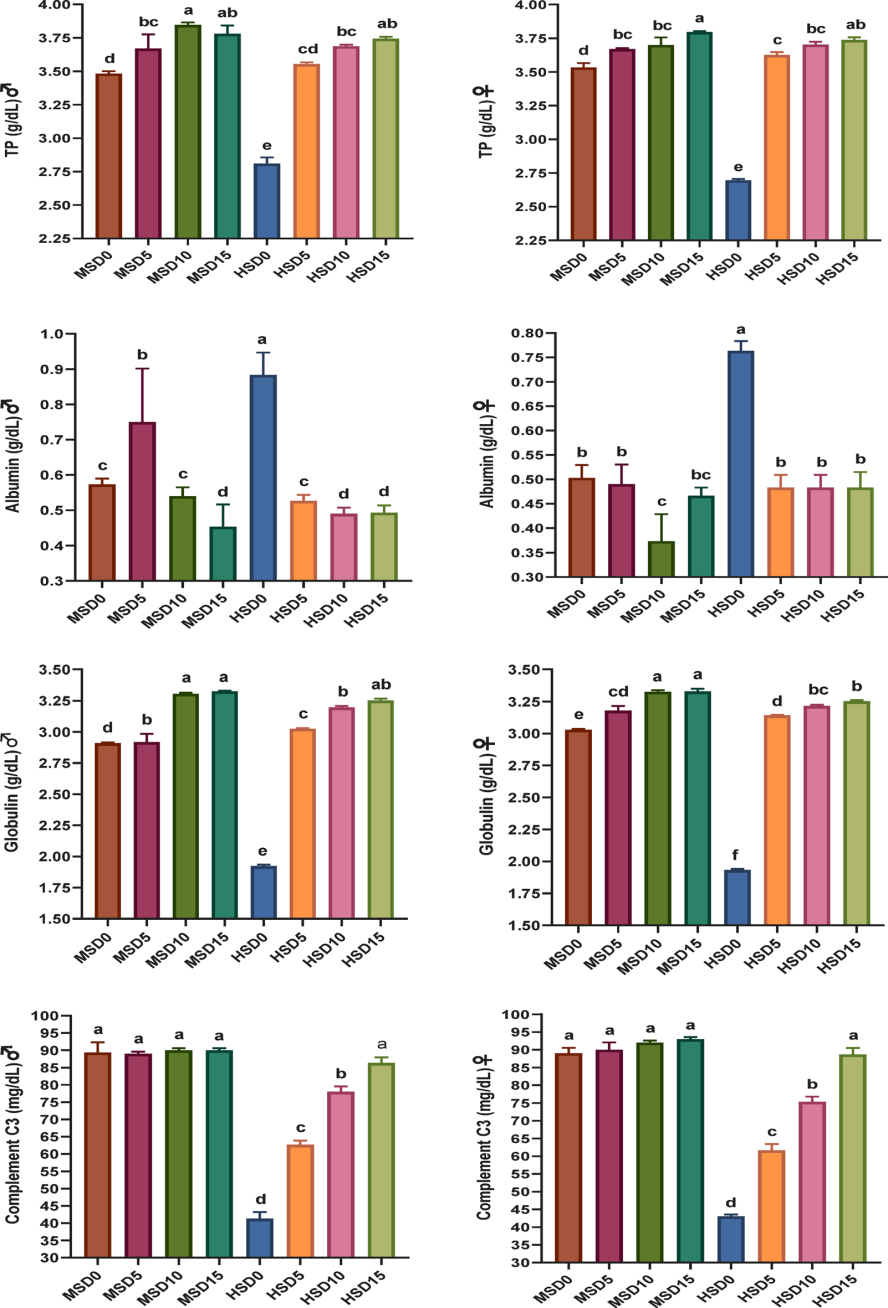

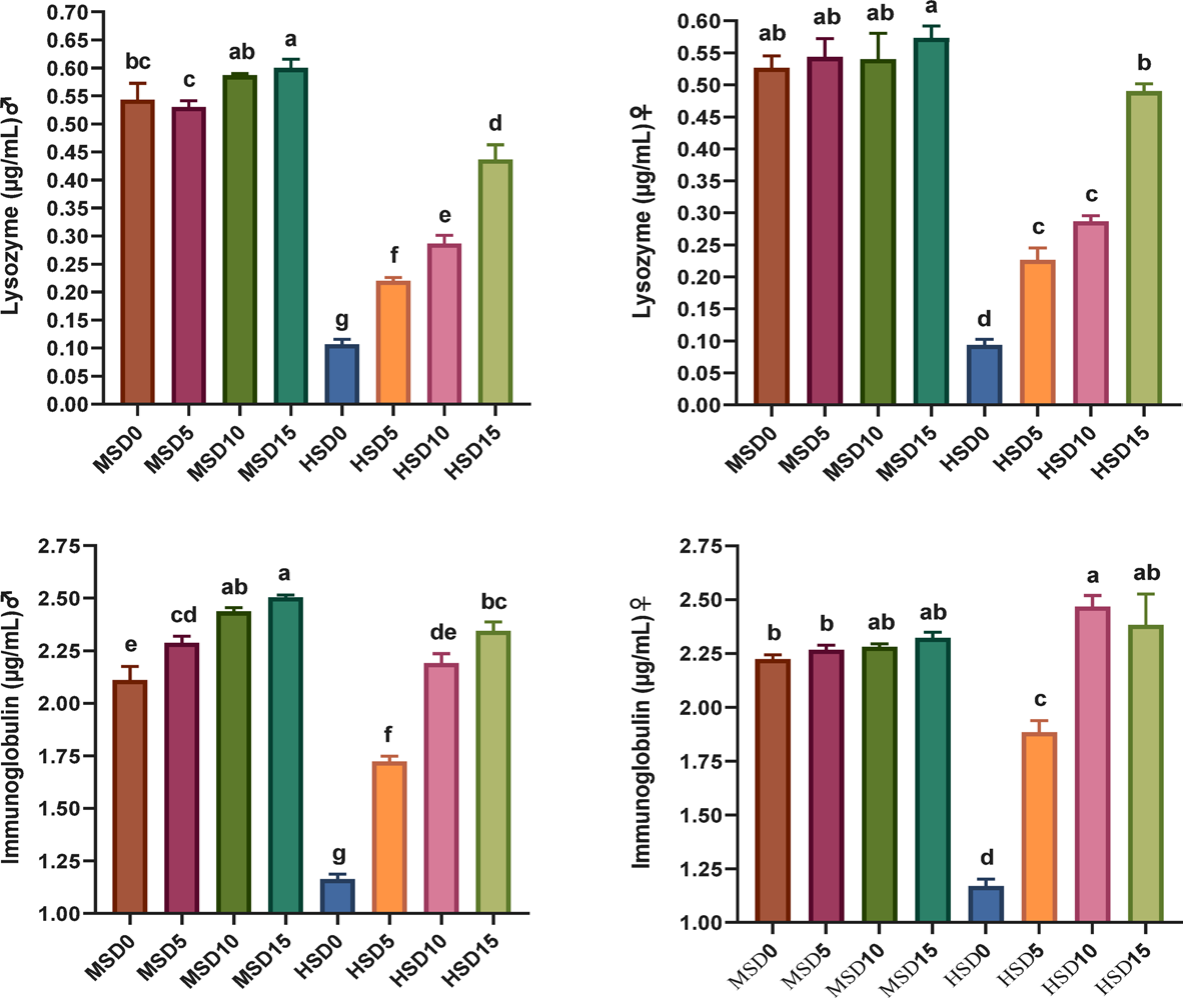
Innate immune markers activity of Florida red tilapia (♂ and ♀) fed Moringa oleifera leaf ethanol extract (MOLE) for 60 days pre-spawning and 120 days spawning period, at different salinities. TP = total protein; IgM = immunoglobin; MSD0 = fish fed D0 diet @ 18ppt salinity; MSD5 = fish fed D5 diet @ 18ppt salinity; MSD10 = fish fed D10 diet @ 18ppt salinity; MSD15 = fish fed D15 diet @ 18ppt salinity; HSD0 = fish fed D0 diet @ 32 ppt salinity; HSD5 = fish fed D5 diet @ 32 ppt salinity; HSD10 = fish fed D10 diet @ 32 ppt salinity; HSD15 = fish fed D15 diet @ 32 ppt salinity; D0 = basal diet without MOLE-supplementation (control); D5 = MOLE-supplemented diet with 5 g/kg; D10 = MOLE-supplemented diet with 10 g/kg; D15 = MOLE-supplemented diet with 15 g/kg.

**Fig. 3:**
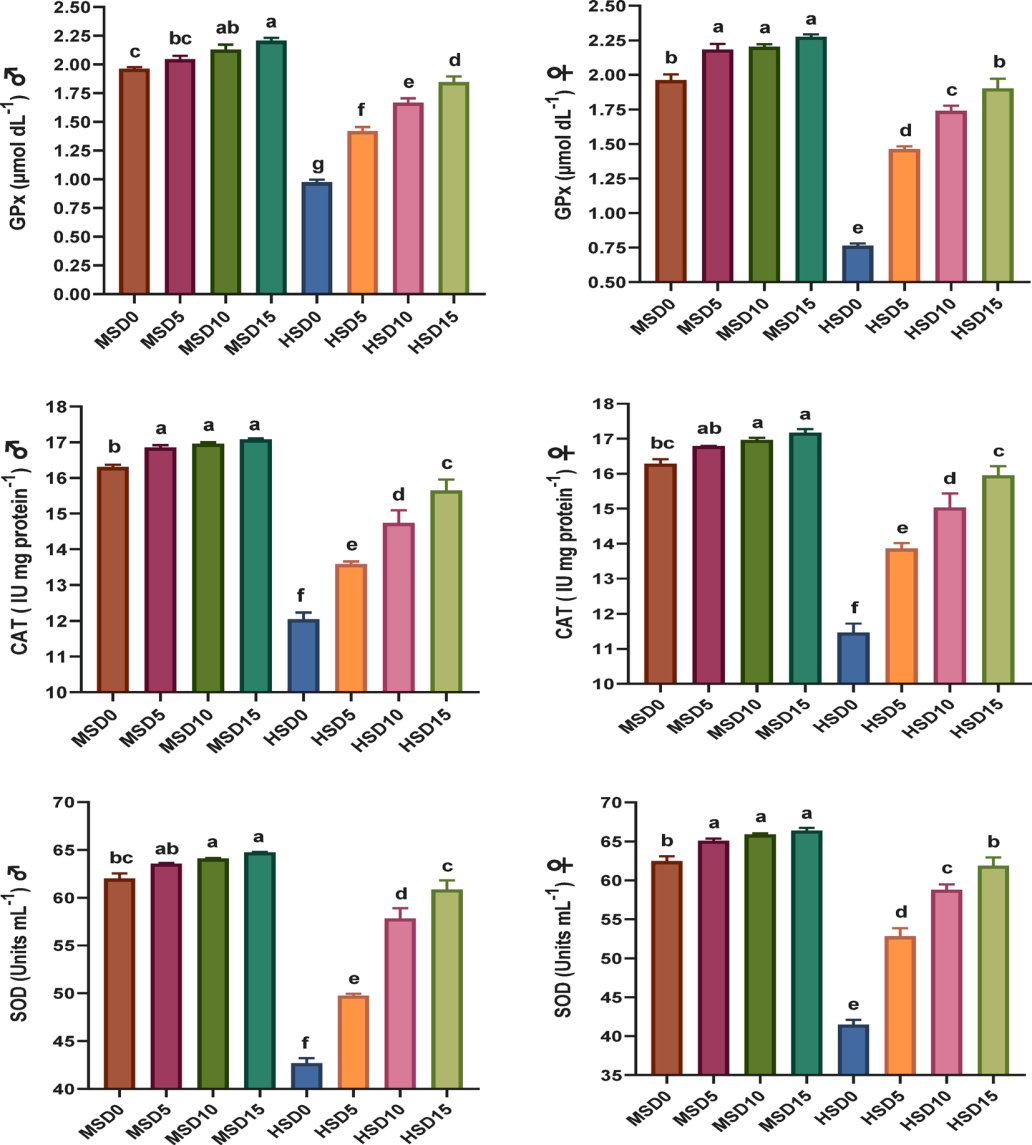

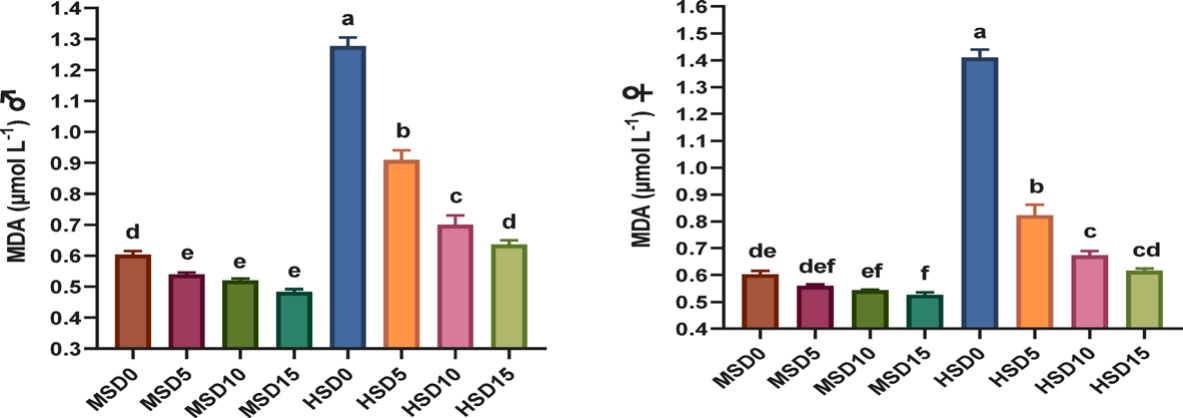
Antioxidative parameters in Florida red tilapia (♂ and ♀) fed Moringa oleifera leaf ethanol extract (MOLE) for 60 days pre-spawning and 120 days spawning period, across different salinities. GPx = glutathione peroxidase; CAT = catalase; SOD = superoxide dismutase; MDA = Malondialdehyde; MSD0 = fish fed D0 diet @ 18ppt salinity; MSD5 = fish fed D5 diet @ 18ppt salinity; MSD10 = fish fed D10 diet @ 18ppt salinity; MSD15 = fish fed D15 diet @ 18ppt salinity; HSD0 = fish fed D0 diet @ 32 ppt salinity; HSD5 = fish fed D5 diet @ 32 ppt salinity; HSD10 = fish fed D10 diet @ 32 ppt salinity; HSD15 = fish fed D15 diet @ 32 ppt salinity; D0 = basal diet without MOLE-supplementation (control); D5 = MOLE-supplemented diet with 5 g/kg; D10 = MOLE-supplemented diet with 10 g/kg; D15 = MOLE-supplemented diet with 15 g/kg; GPx = glutathione peroxidase; CAT = catalase; SOD = superoxide dismutase; MAD = malondialdehyde.

Non-specific immune markers and antioxidant status in FRT (♂ and ♀) fed MOLE-supplemented diets across diverse salinity levels are presented in Figs. 2 and 3, respectively. MOLE doses, salinity, and their interaction significantly affected the activity of most immune markers. The MOLE addition amplified TP and GLO in both sexes under both salinities, IgM and LYZ in ♂ under both salinities, as well as LYZ, IgM and C3 in both sexes at HS of 32 ppt compared to the control group. Furthermore, the inclusion of MOLE enhanced antioxidant (SOD, CAT, and GPx) capacity and diminished MDA activity in both sexes across both salinities compared to the control diet (Fig. 3). All immune and antioxidant markers were significantly affected by salinity, but the effects varied across factors.

### Reproduction and fecundity

**Fig. 4:**
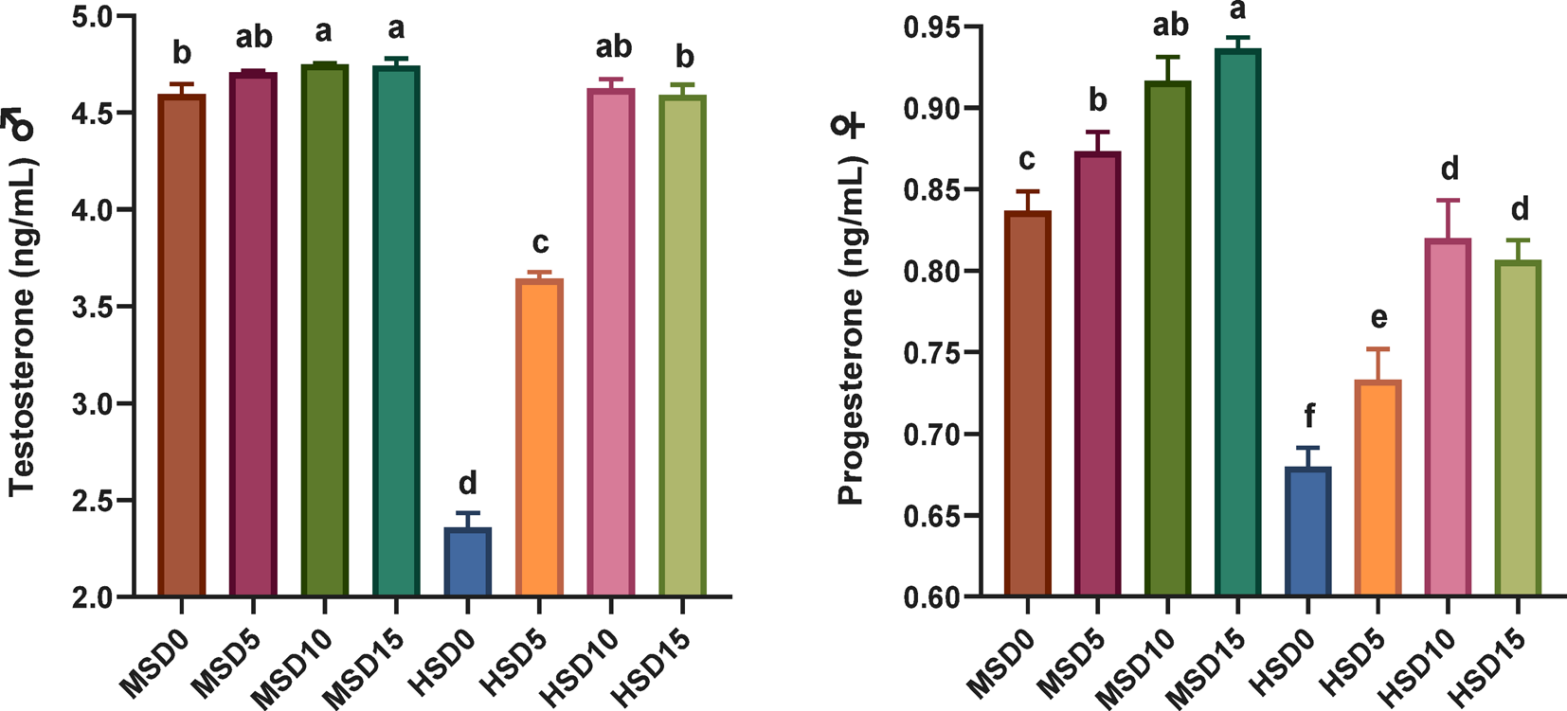
Reproductive hormones **(**testosterone, ng/ml and progesterone, ng/ml) in Florida red tilapia (♂ and ♀) fed Moringa oleifera leaf ethanol extract (MOLE) for 60 days pre-spawning and 120 days spawning period, across different salinities. MSD0 = fish fed D0 diet @ 18ppt salinity; MSD5 = fish fed D5 diet @ 18ppt salinity; MSD10 = fish fed D10 diet @ 18ppt salinity; MSD15 = fish fed D15 diet @ 18ppt salinity; HSD0 = fish fed D0 diet @ 32 ppt salinity; HSD5 = fish fed D5 diet @ 32 ppt salinity; HSD10 = fish fed D10 diet @ 32 ppt salinity; HSD15 = fish fed D15 diet @ 32 ppt salinity; D0 = basal diet without MOLE-supplementation (control); D5 = MOLE-supplemented diet with 5 g/kg; D10 = MOLE-supplemented diet with 10 g/kg; D15 = MOLE-supplemented diet with 15 g/kg.

**Fig. 5:**
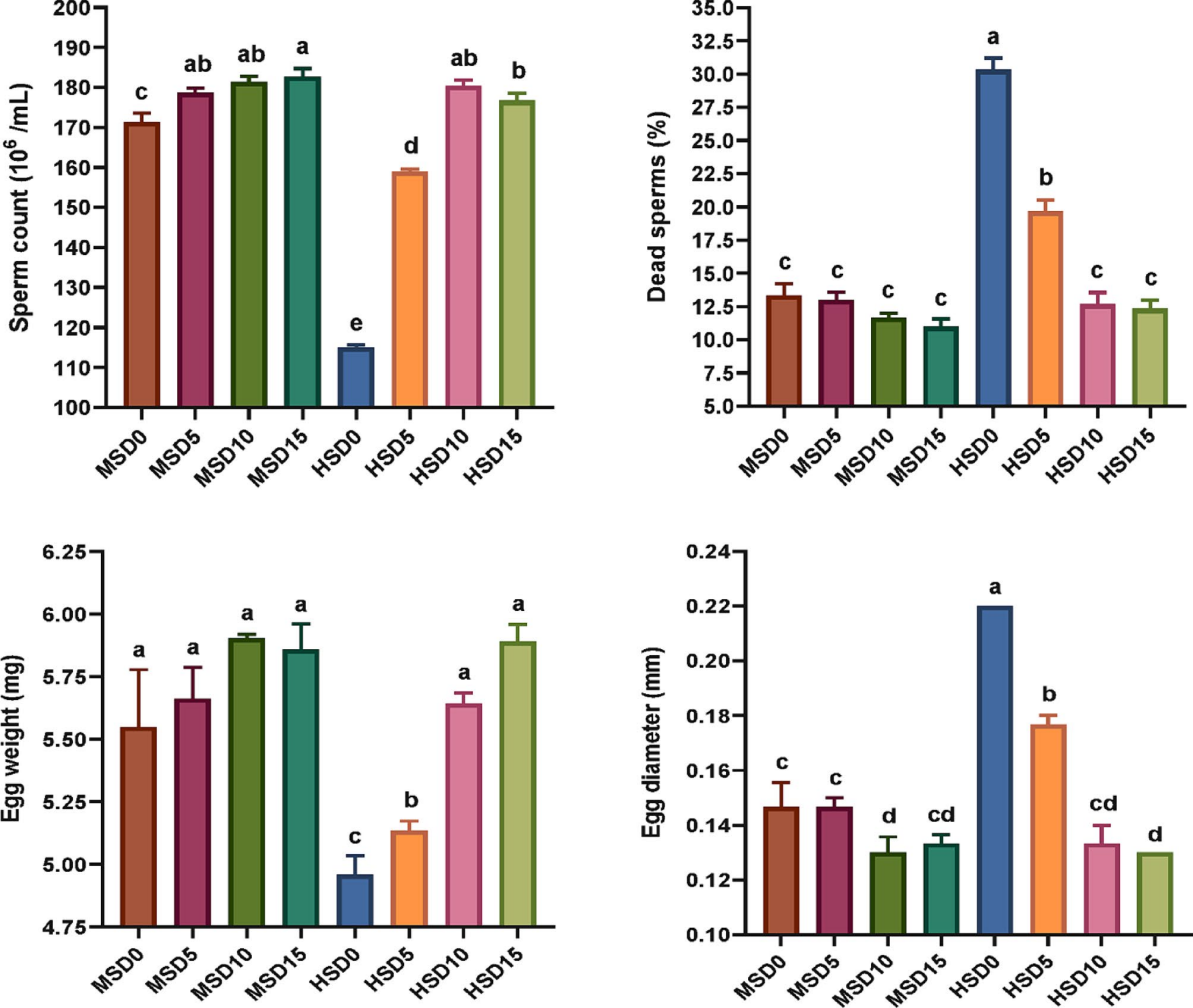
Vitality and characteristics of eggs and sperm of Florida red tilapia (♂ and ♀) fed Moringa oleifera leaf ethanol extract (MOLE) for 60 days pre-spawning and 120 days spawning period, across different salinities. MSD0 = fish fed D0 diet @ 18ppt salinity; MSD5 = fish fed D5 diet @ 18ppt salinity; MSD10 = fish fed D10 diet @ 18ppt salinity; MSD15 = fish fed D15 diet @ 18ppt salinity; HSD0 = fish fed D0 diet @ 32 ppt salinity; HSD5 = fish fed D5 diet @ 32 ppt salinity; HSD10 = fish fed D10 diet @ 32 ppt salinity; HSD15 = fish fed D15 diet @ 32 ppt salinity; D0 = basal diet without MOLE-supplementation (control); D5 = MOLE-supplemented diet with 5 g/kg; D10 = MOLE-supplemented diet with 10 g/kg; D15 = MOLE-supplemented diet with 15 g/kg.

**Fig. 6:**
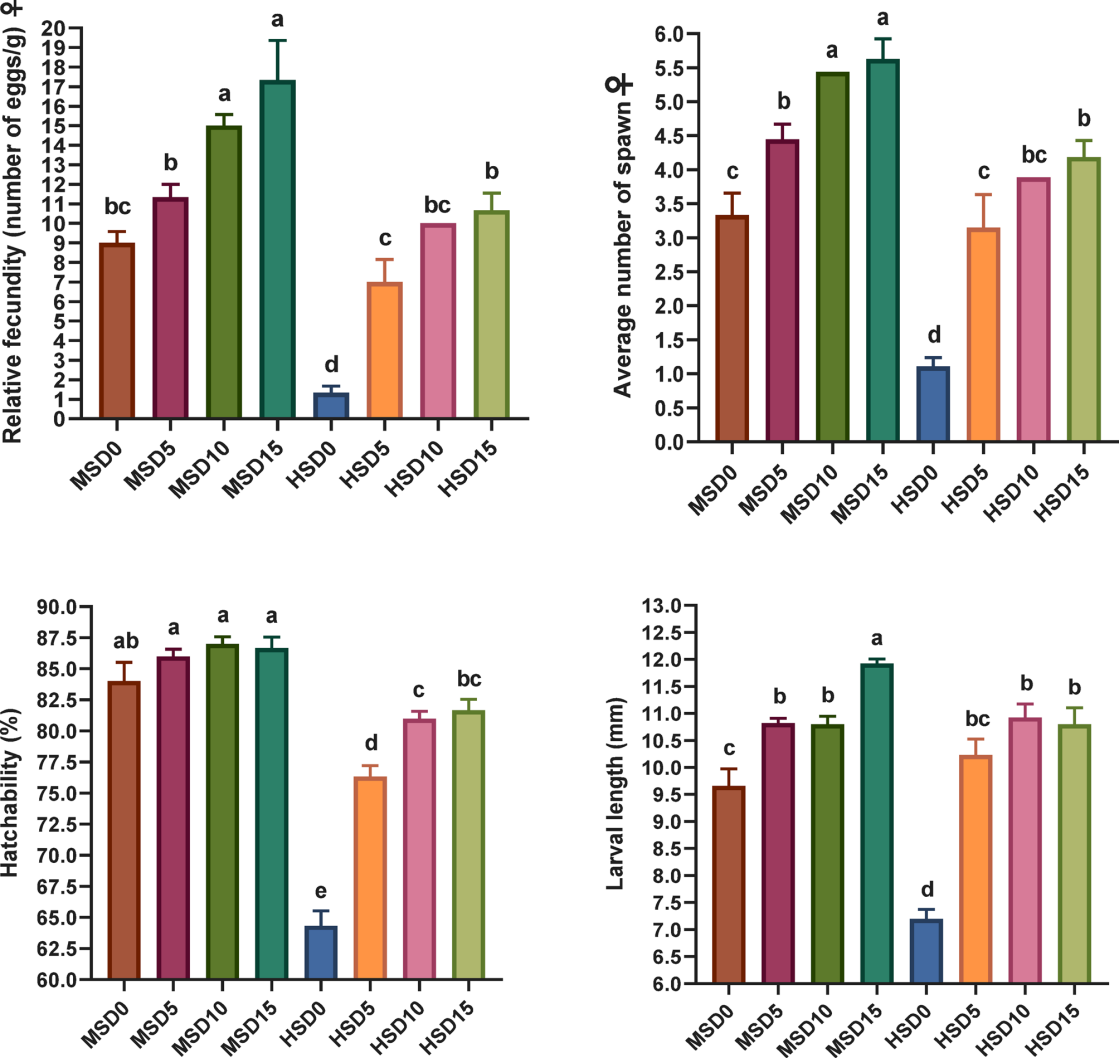
Hatchability, relative fecundity (number of eggs per 1 g), and larval length of Florida red tilapia (♂ and ♀) fed Moringa oleifera leaf ethanol extract (MOLE) for 60 days pre-spawning and 120 days spawning period, across different salinities. MSD0 = fish fed D0 diet @ 18ppt salinity; MSD5 = fish fed D5 diet @ 18ppt salinity; MSD10 = fish fed D10 diet @ 18ppt salinity; MSD15 = fish fed D15 diet @ 18ppt salinity; HSD0 = fish fed D0 diet @ 32 ppt salinity; HSD5 = fish fed D5 diet @ 32 ppt salinity; HSD10 = fish fed D10 diet @ 32 ppt salinity; HSD15 = fish fed D15 diet @ 32 ppt salinity; D0 = basal diet without MOLE-supplementation (control); D5 = MOLE-supplemented diet with 5 g/kg; D10 = MOLE-supplemented diet with 10 g/kg; D15 = MOLE-supplemented diet with 15 g/kg.

The profound effects of high salinity on sex hormones diminishing (Fig. 4), egg and sperm vitality (Fig. 5), hatchability and fecundity (Fig. 6) and overall reproductive performance in FRT and the potential improvements of MOLE addition (Table 10) were demonstrated. Both different salinity and MOLE levels considerably influenced most of the measured factors. Notably, fish fed the MOLE diets exhibited improved reproductive hormones (testosterone and progesterone), sperm count and egg weight, decreased percentage of dead sperm, increased egg production, and increased absolute fecundity, compared to those fed a control diet at both salinities. Salinity alone showed a profound effect associated with decreased measures of reproductive performance, including testosterone, progesterone, sperm count, egg weight, fertility, and increased dead sperm at high salinity. Interestingly, MOLE-salinity interactions improved almost all reproductive performance parameters at both salinities, with the interactive impact of MOLE and MS (18 ppt) being better than that of MOLE and HS (32 ppt). Compared with the MSD0 group (fed a control diet at 18 ppt), administration of MOLE (10–15 g/kg) at HS (32 ppt) enhanced the reproductive performance of FRT, regarding the number of spawning ♀, average spawn times per female, total number of eggs, sperm count, average absolute fecundity, and inter-spawning intervals. Moreover, this resulted in maintaining the same percentage of testosterone and progesterone hormones, the average number of eggs per female, the average days to hatch, and hatchability.

Table 11 illustrates the effects on the chemical composition of eggs of FRT (♀) fed MOLE for 60 days before spawning and 120 days during spawning at different salinities. The administration of MOLE considerably (*p* < 0.05) affected egg analysis, with alterations in eggs’ chemical composition in response to a dose-dependent interaction of MOLE with salinity. Compared to the control diet, fish fed the MOLE diets presented increased protein content and reduced lipid and ash contents under both salinities. Salinity alone noticeably affected the measured contents, resulting in lower protein content and higher lipid and ash content in HS (32 ppt) compared to MS (18 ppt).

## Discussion

Herbal derivatives are useful nutritional supplements in aquaculture^94^. Using the advantages of *M. oleifera*, this research attempts to fill the gap in the poor reproductive function and overall performance of FRT broodstock under full saline conditions.

In this study, there was a considerable enhancement in water quality parameters in MOLE-fed fish compared to those fed a control diet as evidenced by a decrease in nitrogen by-products (NH_4_+, NO_3_^−^, and NO_2_^−^). This result is consistent with the documented effect of MOLE on ammonia (NH_3_) reduction in an earlier study on Nile tilapia^51^. In line with these outcomes, the dietary inclusion of 0.5% MOLE counteracted the ammonia stress in *M. rosenbergii*^44^. In the same vein, previous researchers have demonstrated the ability of *M. oleifera* extract to mitigate some physical stressors, such as heat stress^46^ and starvation stress in Nile tilapia^53^ and captive stress in common carp^95^. These improvements can be connected to enhanced protein metabolism and hence metabolic processes in the fish body, resulting in reduced NH_3_ excretion and diminished accumulation of nitrogen by-products in rearing tanks^96,97^. Moreover, the decreased nitrogenous compounds in MOLE fish can be explained by MOLE having a role in promoting the utilization of dietary protein in fish growth rather than any other metabolic process due to flavonoids and phenolic acid compounds, which have antibacterial, antioxidative, antifungal, and anti-inflammatory effects and selectively increase the probiotic and hinder the growth of pathogenic bacteria^43,98,99^. Furthermore, the role of moringa in alleviating the effect of numerous physical stress improves the fish environment and culture conditions leading to reduced ammonia secretion^41^.

The current investigation revealed significant improvements in growth parameters (FBW, WG, ADG, SGR, and CF) and feed utilization indices (feed intake, FCR, and PER) in FRT broodstocks (♂ and ♀) supplemented with MOLE under both MS (18 ppt) and HS (32 ppt) conditions. These enhancements showed statistically significant interactions between MOLE doses and salinity levels for most growth parameters, except for FBW, WG, ADG, and SGR, in ♀ fed 5 g/kg MOLE under 18 ppt salinity. In addition, interactions between MOLE doses and salinity conditions resulted in significant improvements in most feed efficiency indices except for EU (♀) at both salinities and PPV and EU in ♂ fed 5 g/kg MOLE under 18 ppt salinity. Herein, FRT raised at HS (32 ppt) recorded significant improvements in growth indices when supplemented with MOLE, with ADG increasing by more than 42% and 68% to 0.77 g and 1.03 g in fish fed 15 g/kg MOLE compared to 0.54 g and 0.63 g with those fed a control diet in ♀ and ♂, respectively. Moreover, the FCR improved by more than 38% and 53%, recording 1.21 g and 1.08 g for fish fed 15 g/kg MOLE compared to 1.98 g and 2.33 g in those fed a control diet in ♀ and ♂, respectively. This enhancement can assumably be assigned to MOLE’s multifaceted features. It can mitigate physical stresses (such as hyperthermia, starvation, ammonia, oxidative stress, and high salinity), attenuate signs of toxicity, reduce inflammation, and promote digestion and metabolism, all of which can take part in more active energy use for fish growth as reported in earlier investigations^41,53,98,100^. These results agree with prior studies on moringa extract in various fish species, including *O. niloticus*^51–53^*M. rosenbergii*^44^ and *Cirrhinus mrigala*^54^. Higher salinity may disrupt several physiological functions, such as appetite suppression, growth retardation, and reduced feed utilization^22,101^. Also, osmoregulatory pressure initiates declined feed intake to preserve energy, while metabolic modifications redirect resources out from growth^22^. However, considerable enhancements in feed efficacy indices were marked in FRT supplemented with MOLE across diverse salinity conditions. Both ♀ and ♂ exhibited a decline in feed intake at HS (32 ppt), while the FCR enhanced at both salinity levels. Remarkably, the interaction between MOLE and salinity conditions greatly affected feed utilization (*p* < *0.05*), emphasizing the complicated connection between these factors. Interestingly, the interaction between MOLE at 15 g/kg and HS (32 ppt) resulted in the most desirable values for PER in ♂ and FCR in both sexes, while PER in ♀ favored the interaction between MS (18 ppt) and MOLE at 15 g/kg. Studies propose that MOLE contains phenolic substances that can promote probiotic growth and hinder pathogenic bacteria^102^ and polysaccharides that can modify the intestinal microbial community and its excretion^98^. By facilitating the blossoming of beneficial bacteria in the gut, it likely controls the multiplication of pathogenic microorganisms^98,103^. This approach could clarify the marked upsurge in feed efficacy and possibly the enhanced growth indicators noticed in MOLE-supplemented fish. Additional endowing proof arrives from El-Son, et al.^52^Elabd, et al.^53^ and Mahmoud, et al.^51^ who heeded improved growth parameters in *O. niloticus* fed a moringa-supplemented diet. These researchers attributed these improvements to enhanced digestive enzyme activity, improved intestinal morphology (villus width, villus length, and goblet cell count), and the fact that moringa is rich in essential nutrients, including proteins, essential amino acids, vitamins, minerals, alkaloids, flavonoids, carotenoids, and terpenoids.

The inclusion of MOLE significantly modified the carcass composition in FRT (♂ and ♀). Notably, the interaction between MS (18 ppt) and MOLE doses did not change the moisture content (♀) in all MOLE groups compared to the control group. In contrast, the inclusion of MOLE resulted in higher amounts of moisture, protein, and ash, while reducing fat content in both sexes under both salinity conditions. The significance of ash content in regulating physiological homeostasis is shown by its crucial function in the management of osmotic ions after fish exposure to salinity stress^104^. The cyclical needs of spermatogenesis and ovarian maturation during breeding seasons can cause changes in ash content^105^. In addition, water salinity and mineral abundance can impact mineral adsorption, affecting total ash levels. The elevated moisture, ash, and protein contents and lower lipid content in the present study are in complete alignment with those reported for Nile tilapia-fed moringa extract^51^. Comparable results were collected in *Labeo rohita* by Arsalan, et al.^106^ and *Lates calcarifer* Ganzon-Naret^107^. The high crude protein and ash contents in fish muscle can be attributed to the high levels of protein content (260 g/kg dry matter) and minerals (e.g., calcium, iron, and potassium) in moringa leaves^41,108^. Further, phyto-derivative-based supplemental diets have been demonstrated to improve ash content and diminish lipid levels in marine organisms^22,109^. Likewise, MOLE noticeably influenced organ indices in FRT. In both sexes, HSI and VSI were decreased in fish-fed MOLE compared to those fed a control diet at both salinities. Salinity alone significantly impacted interior organs resulting in increased VSI and HSI in both ♂ and ♀ under HS (32 ppt) compared to MS (18 ppt). Notably, the interaction between MOLE doses and salinity levels remarkably affected gonad and testicular weights (*p* < 0.05), resulting in an increased TSI in ♂ and GSI in ♀ fed MOLE diets compared to the control group. The improvement in reproductive organs can be attributed to the overall improvement in fish performance and health condition due to feeding the fish with MOLE (Miracle tree extract)^41,110^. These findings are compatible with previous investigations demonstrating that African catfish *Clarias gariepinus*, a diverse moringa extract of up to 15%, displayed improvements in ovary weight (♀) and testes weight (♂)^110,111^. Moreover, the effects of MOLE on HSI and VSI are similar to those previously reported in other studies^22^.

Investigation of the influence of MOLE on the activity of digestive enzymes in FRT uncovered a notable improvement in all enzyme activities in both sexes across two salinity classes (18 and 32 ppt) in fish augmented with MOLE, proposing improved protein digestibility. However, amylase activity in ♀ fed 5 g/kg of MOLE under both salinities was not significantly changed compared to the control group, indicating a dependent interaction between MOLE dose and fish sex. Increasing the MOLE dose was associated with improved digestive enzyme activity under HS (32 ppt) showing the highest activity of all enzymes in fish fed 15 g/kg MOLE (HSD15 group). The interaction between optimal MOLE doses and salinity on digestive enzymes in both ♂ and ♀ was demonstrated to be sex-dose-dependent. Salinity stress alone significantly affected the activity of all digestive enzymes, indicating a decrease in HS (32 ppt) compared to MS (18 ppt). The enhanced digestive enzyme activity can be ascribed to the advantageous properties of moringa flavonoids and phenolic acid that can increase beneficial microorganisms in the digestive tract and boost the growth of some probiotic bacteria including such as *L. jonsonii*, *B. adolescentis*, *L. casei*, and *B. lactis*^41,52,98^. Probiotics secrete various enzymes that assist organisms digest dietary components and produce nutrients involved in growth animal while others have antibacterial, antifungal and anti-inflammatory effects that improve intestinal absorption of digested food^41,52,98^. Furthermore, moringa is rich in important amino acids^112^ vitamins (including vitamins A, C, and E), and minerals (such as calcium, iron, and potassium)^108^ that may enhance digestive enzymes and promote feed digestion^41,45,98^. In agreement with the current results, earlier investigations have demonstrated increased digestive enzyme activity in fish supplemented with moringa extracts. For instance, Nile tilapia fed moringa protein hydrolysate^45^ and *Penaeus vannamei* fed moringa leaf extract^98^ showed elevated enzyme levels compared to those fed a control diet.

Screening biochemical indices in fish, including CHO, COR, AST, ALT, urea, and uric acid, provides practical insight into their health status and physiology^113^. CHO, for example, is a crucial component of the biomembrane and the outer layer of blood lipoproteins^114^. On the other hand, ALT and AST serve as metrics for assessing liver health^115^. Urea assessment reflects nitrogen excretion efficiency, and uric acid is associated with metabolic processes^22^while COR is a hormonal marker of stress^116^. Surveying these markers allows for assessing fish well-being and identifying conceivable stresses or infections. This investigation shows that fish plasma indicators depend on sex and dosage in both ♂ and ♀ fish undergoing FRT augmented with MOLE at salinities of 18 and 32 ppt. These alterations are most enunciated in CHO, AST, ALT, COR (♀), and uric acid (♂). The interaction between the ideal dosage of MOLE and salinity significantly impacts most indicators (p *< 0.05*), with the exception of urea (♂) when using 5 g/kg MOLE and COR (♂) when using 10 and 15 g/kg MOLE at 18 ppt, as well as urea and uric acid (♀) and COR (♂) with 5 g/kg MOLE at 32 ppt. These enhancements may be due to MOLE’s hepatoprotective possessions as documented in earlier investigations^46,52,61^. In line with these outcomes, Nile tilapia fed Moringa leaf meal up to 1.5% displayed significant declines in plasma ALT and AST levels and renal function indices (uric acid, urea, and creatinine) compared to controls^117^. Also, MOLE supplementation was shown to control *Aeromonas hydrophila* infections and transport-induced stress leading to significant reductions in AST, ALT lactate dehydrogenase, malate dehydrogenase, cortisol and glucose (P < *0.05*) in Nile tilapia fed augmented diets^46^. Moreover, MOLE supplementation reduced the effect of sub‑lethal fipronil in Nile tilapia and appeased hepato-renal failure (increased urea, creatinine, ALT, and AST)^51^. Furthermore, MOLE inclusion regulated the physiological response of *M. rosenbergii* to *Vibrio anguillarum* and ammonia stress and significantly lessened ALT and AST levels^44^. The authors linked the hepatoprotective effect of moringa leaves to their ability to scavenge free radicals, maintain liver cell membrane integrity, increase antioxidant enzyme activities, and prevent reactive oxygen species damage^41,44,46^.

Scrutinizing blood biochemistry in fish delivers practical insights into their welfare, immune-physiological condition, nutritional values of diets and observing the impacts of harmful agents^41,113^. A robust innate immune status is linked to elevated levels of TP, ALB, GLO, and IgM, which describe the main proteins in fish serum^6^. Moreover, leukocytes have a crucial part in the innate immune system upon inflammation, and their numbers are an indicator of the health status of fish^118,119^. In addition, a higher level of red blood cell (RBC) count reflects a healthy physiological condition, which is represented in growth performance^120,121^. Our findings here reveal dose-dependent improvements in plasma immune parameters of FRT (♀ and ♂) augmented with MOLE at 18 and 32 ppt salinities. These enhancements are most conspicuous in TP and GLO in both sexes and IgM (♂) under both salinities (18 and 32 ppt), LYZ, IgM, and C3 (♀) at HS (32 ppt), and LYZ and C3 (♂) at HS (32 ppt). Furthermore, fish fed MOLE-supplemented diets showed an overall increase in RBCs and WBCs (neutrophils, lymphocytes, and monocytes) numbers compared to those fed a control diet, even if some parameters remained non-significantly variable with the control group. Notably, the interaction between optimal MOLE dose and salinity remarkably enhanced most innate immune indicators (*p* < *0.05*) in both sexes, except for ALB in all conditions and LYZ and IgM in ♀ at MS (18 ppt). Earlier studies have documented the stimulating effect of moringa extracts on the innate immune system in aquatic animals. For example, Mansour, et al.^122^ documented a remarkable improvement in systemic immunity of *S. aurata* through elevated phagocytic ability of head kidney leucocytes, peroxidase, and respiratory burst activities alongside improved plasma LYZ, IgM, and alternative complement pathway activity. Furthermore, El-Son, et al.^52^ reported increased LYZ, IgM, and C3 activity in Nile tilapia fed MOLE-fortified diets compared to those fed the control. Also, moringa leaf powder administration significantly increased the hematological parameters (i.e., RBCs, WBCs counts, and PCV, Hb, MCV, MCH and MCHC)^53^ and improved the immune response (i.e., respiratory burst, phagocytic, LYZ, and IgM activities and WBC numbers) in Nile tilapia fry exposed to *Aeromonas hydrophila*^123^.

Besides, the amplified immune potency in fish-fed MOLE diets, the antioxidant capacity in such fish was also greater than that of those fed a control diet. SOD, CAT, and GPx are the main antioxidant enzymes in fish bodies that may dispose of unwanted O_2_ − and H_2_O_2_, and ROOH generated by free radicals^85,124^. Our findings demonstrate that MOLE augmentation considerably improves antioxidant enzymes in FRT broodstock at both HS and MS, with all fish (♂ and ♀) fed varying MOLE doses under HS displaying decreased MDA and increased antioxidant enzymes (SOD, CAT, and GPx). This marked synergy between MOLE doses and salinity considerably improved antioxidant enzymes (*p* < *0.05*), more pronounced under HS (32 ppt). These results are consistent with prior studies on MOLE’s capacity to boost antioxidant activity in diverse fishes, such as Nile tilapia^46,52,53^ and *S. aurata*^61^ as well as crustaceans including *Penaeus vannamei*^98^ and *M. rosenbergii*^44^. The researchers attributed these improvements in fish immunity and antioxidant capacity to the richness of moringa in bioactive components such as polyphenolic compounds, volatile oils, vitamins, minerals, phenolic acids, flavonoids, carotenoids, tannins, saponins, alkaloids, anthocyanins, terpenoids, β-sitosterol, anthraquinone, coumarins, proanthocyanins, isothiocyanate and glycoside cyanides that can stimulate the immune system of fish and boost their antioxidative capacity^41,52,61,98^.

Salinity and feed quality play a key role in fish reproduction, spotlighting their impact through salinity sufferance and diet ingredients^37^. Sokolova, et al.^125^ documented an increase in energy requirements upon moving from lower to higher salinity, affecting all aspects of fish performance, including reproduction. Furthermore, a general inhibitory role of salinity on tilapia reproduction has been reported with increasing salinity degrees^39^. Engdaw and Geremew^38^ emphasize the decisive role of specific and quantitative energy input in providing adequate nutrition for osmotic pressure regulation and successful reproduction. Despite various tilapia species having salt-tolerant genes, they have shown diminished embryo production under saline conditions. Thus, improving FRT proliferation requires apprehending the crucial aspects that affect this paramount physiological process. As for FRT in particular, increasing salinity above 18 ppt, considerably declines seed production per unit weight of female, proposing a possible controversy in the balance of energy allocation to hatching and osmoregulation^21^.

Sex steroids play an important role in many basic physiological processes in all vertebrates^57,126^. Estimating blood levels of sex hormones appears to be a beneficial tool for assessing the reproductive cycle of fish^127,128^. Testosterone is created by Leydig cells, which play a major part in sperm formation and the development of secondary sexual characteristics in ♂. Progestins are major regulators of spawning in fish and vertebrates^129^. Progesterone is a pivotal mediator in the biosynthesis of many active steroids in fish, comprising androgens such as estradiol-17a, estradiol-17b, and 20b-dihydroxyprogesterone (17a, 20b-P)^129,130^. In this study, excluding fish fed 5 g/kg MOLE at MS (18 ppt), FRT broodstock fed MOLE diets displayed higher testosterone and progesterone activity than those fed the control diet under salinity levels of 18 and 32 ppt. Interestingly, fish fed 10 g/kg MOLE at 32 ppt salinity showed reproductive hormone activity similar to their counterparts fed the control diet at 18 ppt salinity. Furthermore, increasing the MOLE dose to 15 g/kg did not improve testosterone and progesterone activity at 32 ppt salinity, in contrast to what occurred at 18 ppt salinity. This can be attributed to the interplay between salinity and MOLE dose, which varies from one salinity level to another and affects the biological and physiological needs of fish. These results are consistent with the improved estradiol and testosterone concentrations detected in Nile tilapia fed 0.5 g/kg^56^ and 200 mg/kg^57^ of *M. oleifera*. The researchers attributed this improvement to the high content of unsaturated fatty acids (in particular linolenic acid) in *M. oleifera*.

Notably, the current examination reveals that MOLE augmentation positively influences FRT’s reproductive activity under saline water, improving most parameters. Interestingly, ♀ fed 10 g/kg MOLE at 32 ppt salinity showed similarity to those fed the control diet at 18 ppt salinity, regarding inter-spawning intervals (days), number of spawning ♀/groups, total number of eggs per ♀, fecundity, and average days to hatch, while outperforming in larval length (mm). In addition, these ♀ (fed 10 g/kg MOLE at 32 ppt salinity) showed comparable values ​for average days to hatch/group, larval length, and average number of eggs per female with those fed the same dose at 18 ppt salinity, while they were superior in decreased time to first oviposition (days) and inter-ovulation intervals (days). Furthermore, ♀ of 10 g/kg MOLE at 32 ppt salinity maintained the same reproductive performance indicators as those fed 15 g/kg MOLE at the same salinity condition, except for the total number of eggs per ♀ and hatchability. Salinity alone adversely impacted FRT reproductive function showing the worst results in fish fed a control diet at 32 ppt salinity. Furthermore, a considerable interplay between MOLE doses and salinity levels was marked on sex hormones (♂ and ♀), females’ fertility indices, and spawn egg composition, indicating a complicated interaction between the dose, salinity, and the consequent reproductive result. Changes in salinity levels may necessitate higher energy expenditure to sustain the equilibrium of bodily fluids, particularly in the control of sodium and chloride ions^30^. This may explain the difference in fecundity of FRT fed the same doses of MOLE under varying salinity conditions. In this regard, no previous experiences have been documented on the combined effect of dietary *M. oleifera* and high salinity on fish reproduction to compare our results with. However, in line with these outcomes, Nile tilapia fed *M. oleifera* extract showed improved gonadal histo-architecture and reproductive performance^57^ as well as increased sex hormone activity and GSI (♂ and ♀) along with the number of fries produced when fed *M. oleifera* seed extract^56^. Also, Paul, et al.^131^ revealed that the nutritional content of moringa is adequate to sustain and improve the quality of reproductive performance and general health in zebrafish.

In the same vein, numerous studies have involved diet as an influential facet impacting spermatogenesis, sperm and ovary maturation, and fertilizing capability^132^. Subsidizing the existing findings, it was stated that moringa leaves affect reproductive actions in mice and sows^133,134^ as well as on the quality of sperm and the levels of testosterone in rats^134,135^. In addition, the consumption of moringa leaves in the diet of male rainbow trout *Oncorhynchus mykiss* revealed enhanced sperm quality and increased reproductive performance^50^. The enhanced reproductive capacities of fish may be attributed to the abundant quantities of micronutrients found in moringa, including vitamins (Vit-A, C, and E) and minerals (Ca, Fe, Zn, and K) that are crucial for the reproductive biology process^136^. The leaves of Moringa plants are rich in zinc, with a concentration of 64 mg per 100 g^137^ This element has been shown to be essential for improving sperm quality indices and reproductive activity in Nile tilapia^138^. Furthermore, moringa leaves are a highly beneficial source of magnesium (448 mg per 100 g)^139^ which has been shown to have a positive correlation with sperm concentration^140^ and selenium (363 mg/kg)^136^ which protects sperm against reactive oxidative stress and supplies essential proteins and enzymes that significantly impact sperm production and male fertility^50,141^. Furthermore, moringa contains beta-carotene, a precursor to vitamin A, which is found in exceptionally high levels in leaves^142^ high crude fat (10.6%), crude protein, polyunsaturated fatty acids, essential amino acids (16 to 19 amino acid), and a significant quantity of flavanol glycosides (e.g., quercetin, kaempferol) and alkaloids that can promote volitional feed intake^41,49,50^ and thus boost reproductive efficacy.

## Conclusion

This work highlights MOLE as a potential natural promoter in aquaculture, providing a plausible key to mitigate the adverse impacts of high salinity on red tilapia performance, immune-physiological response, fertility, reproductive function, and welfare. The research outcomes demonstrate the remarkable advantages of using 10–15 g/kg of MOLE as a dietary complement for FRT broodstock across moderate (18 ppt) and high salinity (32 ppt). Compared with those fed a control diet, groups supplemented with MOEL at 10–15 g/kg exhibited significant enhancements across several parameters, including growth, feed efficacy, digestive enzyme activity, haemato-biochemical indices, antioxidant capacity, innate immunity, inner reproductive organs, sex hormones, female fertility and overall reproductive performance under both salinity levels. Interestingly, the reproductive process in FRT fed 10–15 g/kg MOLE at 32 ppt water salinity was significantly improved to be comparable to those fed a control diet without additives and a diet supplemented with 5 g/kg MOLE at 18 ppt salinity. This confirms the role of MOLE in alleviating the adverse effects of high salinity and enhancing the resilience of FRT against severe environmental conditions.

**Table 1:** Formulation and approximate chemical composition of the experimental diets supplemented with different doses of Moringa oleifera leaf ethanol extract (MOLE).

Ingredients (g/kg)	D0	D5	D10	D15
Moringa oleifera leaf extract (MOLE)	0	5	10	15
Fish meal (600 g/kg CP)	80	80	80	80
shrimp meal (580 g/kg CP)	23	23	23	23
Meat and bone meal (450 g/kg CP)	20	20	20	20
Corn gluten meal (600 g/kg CP)	30	30	30	30
Soybean meal (460 g/kg CP)	335	335	335	335
Sesame seed meal (410 g/kg CP)	40	40	40	40
Wheat middling (120 g/kg CP)	220	215	210	205
Rice polishing (73 g/kg CP)	133	133	133	133
Corn meal (75 g/kg CP)	90	90	90	90
Soybean oil	12	12	12	12
Di Calcium Phosphate	6	6	6	6
Fish Vitamin & Mineral Premix^**1**^	3	3	3	3
DI Methionine	1.25	1.25	1.25	1.25
L Lysine	1	1	1	1
Choline chloride	5	5	5	5
Vitamin C (Stay C)	0.25	0.25	0.25	0.25
Antioxidant (BHT)^**2**^	0.5	0.5	0.5	0.5
Total ingredients	1000	1000	1000	1000

Chemical composition (%)				
Dry matter, DM	91.06	90.91	90.98	90.89
Crude protein, CP	30.68	30.59	30.71	30.73
Lipid	6.51	6.49	6.47	6.44
Ash	8.31	8.35	8.55	8.58
Fiber, CF	6.81	6.79	6.76	6.73
Nitrogen free extract (NFE)^**3**^	38.75	38.69	38.49	38.41
GE (Kcal/kg)^**4**^	3937.5	3928.1	3924.8	3919.8
GE (MJ/kg)^**5**^	16.48	16.44	16.42	16.40

Experimental diets: D0: MOLE non-supplemented diet (control); D5: MOLE-supplemented diet with 5 g/kg; D10: MOLE-supplemented diet with 10 g/kg; D15: MOLE-supplemented diet with 15 g/kg; MOLE = Moringa oleifera leaf ethanol extract^1^. Trace mineral premix and vitamin premix were purchased from Trouw Nutrition Egypt. (https://www.trouwnutrition.com/en/about-us/global-presence/). ^2^BHT = Butylated hydroxytoluene; ^3^NFE % = DM − (fibers % + protein % + lipids % + ash%)^4^. Gross energy, Kcal/kg was calculated using a value of 5.64 Kcal/g proteins, 9.44 Kcal/g fat, and 4.11 Kcal/g carbohydrates (NFE)^5^. kcal = 0.004184595 MJ.

**Table 2:** Experimental design: the pre-spawning period (phase A) lasted for 60 days in 48 pre-spawning tanks, representing 16 groups of broodstock in triplicate, and the spawning period (phase B) lasted for 120 days in 24 spawning tanks, representing 8 groups of broodstock in triplicate.

Treatments	Salinity (ppt)	Diet type	Broodstocks status	
Pre-spawning (phase A, 60 days)			Broodstocks separated by sex in 48 pre-spawning tanks (each tank has 13♂ or13♀) representing 16 groups	
(MSD0)	18	D0 (control, no MOLE)	13 ♂	13 ♀
(MSD5)	18	D5 (MOLE 5 g/kg)	13 ♂	13 ♀
(MSD10)	18	D10 (MOLE 10 g/kg)	13 ♂	13 ♀
(MSD15)	18	D15 (MOLE 15 g/kg)	13 ♂	13 ♀
(HSD0)	32	D0 (control, no MOLE)	13 ♂	13 ♀
(HSD5)	32	D5 (MOLE 5 g/kg)	13 ♂	13 ♀
(HSD10)	32	D10 (MOLE 10 g/kg)	13 ♂	13 ♀
(HSD15)	32	D15 (MOLE 15 g/kg)	13 ♂	13 ♀

Spawning (Phase B,120 days)			Broodstocks collected in the same tank (each tanke has 3♂ + 6♀) representing 8 groups in triplicate	
(MSD0)	18	D0 (control, no MOLE)	3 ♂ +6 ♀	
(MSD5)	18	D5 (MOLE 5 g/kg)	3 ♂ +6 ♀	
(MSD10)	18	D10 (MOLE 10 g/kg)	3 ♂ +6 ♀	
(MSD15)	18	D15 (MOLE 15 g/kg)	3 ♂ +6 ♀	
(HSD0)	32	D0 (control, no MOLE)	3 ♂ +6 ♀	
(HSD5)	32	D5 (MOLE 5 g/kg)	3 ♂ +6 ♀	
(HSD10)	32	D10 (MOLE 10 g/kg)	3 ♂ +6 ♀	
(HSD15)	32	D15 (MOLE 15 g/kg)	3 ♂ +6 ♀	

MOLE = Moringa oleifera leaf ethanol extract; D0 = basal diet without MOLE-supplementation (control); D5 = MOLE-supplemented diet with 5 g/kg; D10 = MOLE-supplemented diet with 10 g/kg; D15 = MOLE-supplemented diet with 15 g/kg; Treatments: MSD0 = fish fed D0 diet @ 18ppt salinity; MSD5 = fish fed D5 diet @ 18ppt salinity; MSD10 = fish fed D10 diet @ 18ppt salinity; MSD15 = fish fed D15 diet @ 18ppt salinity; HSD0 = fish fed D0 diet @ 32 ppt salinity; HSD5 = fish fed D5 diet @ 32 ppt salinity; HSD10 = fish fed D10 diet @ 32 ppt salinity; HSD15 = fish fed D15 diet @ 32 ppt salinity.

**Table 3:** Water quality findings in spawning tanks used to Raise Florida red tilapia fed different levels of Moringa oleifera leaf ethanol extract (MOLE) at different salinities during 120-day spawning periods.

Variable	Salinity	(18ppt)				(32ppt)				*P*-value		
	Diet	D0	D5	D10	D15	D0	D5	D10	D15	Salinity	MOLE	S×M^1^
	Treatment	MSD0	MSD5	MSD10	MSD15	HSD0	HSD5	HSD10	HSD15			
Temperature, °C		26.11 ± 0.73	26.61 ± 0.53	26.55 ± 0.20	26.22 ± 0.16	26.42 ± 0.05	26.19 ± 0.09	26.33 ± 0.13	26.23 ± 0.03	0.006	0.000	0.000
pH		7.23 ± 0.03^d^	7.43 ± 0.01^bc^	7.54 ± 0.08^b^	7.74 ± 0.01^a^	7.50 ± 0.09^b^	7.69 ± 0.02^a^	7.71 ± 0.02^a^	7.36 ± 0.02^cd^	0.026	0.000	0.000
DO_2_, ppm		5.68 ± 03^d^	6.07 ± 04^bc^	6.40 ± 0.09^a^	6.32 ± 0.11^a^	5.58 ± 0.10^d^	6.25 ± 0.02 ^ab^	5.96 ± 0.03^c^	5.55 ± 0.03^d^	0.000	0.000	0.000
NH4+, ppm		1.03 ± 0.03^a^	0.92 ± 0.03^b^	0.92 ± 0.05^b^	0.82 ± 0.01^c^	0.86 ± 0.02^bc^	0.71 ± 0.01^d^	0.82 ± 0.00^c^	1.06 ± 0.02^a^	0.003	0.000	0.000
NO2-, ppm		0.023 ± 0.00^ab^	0.021 ± 0.00^abc^	0.019 ± 0.00^bc^	0.013 ± 0.00^de^	0.017 ± 0.00^cd^	0.008 ± 0.00^e^	0.011 ± 0.00^e^	0.024 ± 0.00^a^	0.004	0.014	0.000
NO3-, ppm		0.317 ± 0.01^d^	0.348 ± 0.01^c^	0.350 ± 0.01^bc^	0.386 ± 0.01^a^	0.323 ± 0.00^d^	0.337 ± 0.01^cd^	0.357 ± 0.01^bc^	0.369 ± 0.01^ab^	0.442	0.000	0.216

Means in the same row without a common superscript letter differ significantly (*P <* 0.05) as analyzed by two-way ANOVA and the TUKEY test. MOLE = Moringa oleifera leaf ethanol extract; S × M = Salinity × MOLE interaction effect; D0 = basal diet without MOLE-supplementation (control); D5 = MOLE-supplemented diet with 5 g/kg; D10 = MOLE-supplemented diet with 10 g/kg; D15 = MOLE-supplemented diet with 15 g/kg; Treatments: MSD0 = fish fed D0 diet @ 18ppt salinity; MSD5 = fish fed D5 diet @ 18ppt salinity; MSD10 = fish fed D10 diet @ 18ppt salinity; MSD15 = fish fed D15 diet @ 18ppt salinity; HSD0 = fish fed D0 diet @ 32 ppt salinity; HSD5 = fish fed D5 diet @ 32 ppt salinity; HSD10 = fish fed D10 diet @ 32 ppt salinity; HSD15 = fish fed D15 diet @ 32 ppt salinity; DO2 = Dissolved oxygen; NH4 + = ammonium ion; NO2 = Nitrite; NO3 = Nitrate.

**Table 4:** Growth performance of Florida red tilapia (♂ and ♀) fed Moringa oleifera leaf ethanol extract (MOLE) for 60 days pre-spawning and 120 days spawning period, at different salinities.

Variable	Salinity	(18ppt)				(32ppt)				*P*-value		
	Diet	D0	D5	D10	D15	D0	D5	D10	D15	Salinity	MOLE	S×M
	Treatment	MSD0	MSD5	MSD10	MSD15	HSD0	HSD5	HSD10	HSD15			
Female ♀												
IBW, g		98 ± 0.15	97.9 ± 0.63	97.3 ± 0.36	97.8 ± 0.34	97.1 ± 0.91	98.4 ± 0.64	96.7 ± 0.34	96.5 ± 0.33	0.393	0.553	0.394
FBW, g		141 ± 0.49^b^	140 ± 0.44^b^	145 ± 0.81^ab^	150 ± 1.71^a^	130 ± 0.55^c^	139 ± 1.6^b^	138 ± 0.78^b^	143 ± 2.07^ab^	0.483	0.025	0.04
WG, g/fish/day		43 ± 0.53^c^	42 ± 0.92^c^	47.4 ± 0.69^b^	52.4 ± 1.4^a^	32.6 ± 1.06^d^	40.6 ± 1.06^c^	41.6 ± 0.53^c^	46.2 ± 1.83^b^	0.848	< 0.001	< 0.001
ADG, g/fish/day		0.72 ± 0.01^cd^	0.7 ± 0.02^d^	0.79 ± 0.01^b^	0.87 ± 0.02^a^	0.54 ± 0.02^e^	0.68 ± 0.02^d^	0.69 ± 0.01^d^	0.77 ± 0.03^bc^	0.848	< 0.001	< 0.001
SGR, %/day/fish		0.61 ± 0.01^b^	0.60 ± 0.01^b^	0.66 ± 0.01^ab^	0.72 ± 0.01^a^	0.48 ± 0.02^c^	0.65 ± 0.05^ab^	0.60 ± 0.01^b^	0.652 ± 0.02^ab^	0.39	< 0.001	0.001
RGR, %		144 ± 0.57^b^	143 ± 1.18^b^	149 ± 0.72^ab^	154 ± 1.27^a^	134 ± 1.36^c^	147 ± 4.55^ab^	143 ± 0.47^b^	148 ± 1.8^ab^	0.321	< 0.001	0.002
CF		2.42 ± 0.02^e^	3.11 ± 0.11^c^	3.38 ± 0.07^ab^	3.55 ± 0.00^a^	2.61 ± 0.07^e^	2.86 ± 0.08^d^	3.27 ± 0.13^bc^	3.49 ± 0.03^ab^	0.612	< 0.001	0.05
Male ♂												
IBW, g		123 ± 0.33	123 ± 0.333	123 ± 0.67	123 ± 0.33	123 ± 0.58	123 ± 00	123 ± 0.58	123 ± 0.67	0.811	0.884	0.668
FBW, g		165 ± 0.55^cd^	174 ± 0.74^b^	175 ± 2.55^b^	187 ± 1.68^a^	161 ± 0.57^d^	168 ± 1.91^c^	185 ± 1.79^a^	184 ± 1.95^a^	0.001	< 0.001	0.007
WG, g/fish/day		42.4 ± 0.3^cd^	51.2 ± 0.92^b^	51.6 ± 2.11^b^	63.8 ± 1.59^a^	37.8 ± 0.7^d^	45.0 ± 1.91^c^	61.8 ± 2.16^a^	61.8 ± 2.2^a^	0.001	< 0.001	0.014
ADG, g/fish/day		0.71 ± 0.01^cd^	0.85 ± 0.02^b^	0.68 ± 0.04^b^	1.06 ± 0.03^a^	0.63 ± 0.01^d^	0.75 ± 0.03^c^	1.03 ± 0.04^a^	1.03 ± 0.04^a^	0.001	< 0.001	0.014
SGR, %/day/fish		0.49 ± 0.00^cd^	0.58 ± 0.01^b^	0.58 ± 0.02^b^	0.69 ± 0.01^a^	0.45 ± 0.01^d^	0.52 ± 0.02^c^	0.68 ± 0.02^a^	0.68 ± 0.02^a^	0.001	< 0.001	0.024
Relative GR, %		134 ± 0.22^cd^	142 ± 0.82^b^	142 ± 1.57^b^	152 ± 1.27^a^	131 ± 0.68^d^	137 ± 1.55^c^	150 ± 1.9^a^	150 ± 1.94^a^	0.001	< 0.001	0.023
CF		1.56 ± 0.00^e^	2.2 ± 0.07^b^	2.29 ± 0.04^ab^	2.39 ± 0.01^a^	1.88 ± 0.02^d^	2.04 ± 0.03^c^	2.36 ± 0.10^ab^	2.27 ± 0.05^ab^	0.152	< 0.001	0.001

Means in the same row without a common superscript letter differ significantly (*P <* 0.05) as analyzed by two-way ANOVA and the TUKEY test. MOLE = Moringa oleifera leaf ethanol extract; S × M = Salinity × MOLE interaction effect; D0 = basal diet without MOLE-supplementation (control); D5 = MOLE-supplemented diet with 5 g/kg; D10 = MOLE-supplemented diet with 10 g/kg; D15 = MOLE-supplemented diet with 15 g/kg; Treatments: MSD0 = fish fed D0 diet @ 18ppt salinity; MSD5 = fish fed D5 diet @ 18ppt salinity; MSD10 = fish fed D10 diet @ 18ppt salinity; MSD15 = fish fed D15 diet @ 18ppt salinity; HSD0 = fish fed D0 diet @ 32 ppt salinity; HSD5 = fish fed D5 diet @ 32 ppt salinity; HSD10 = fish fed D10 diet @ 32 ppt salinity; HSD15 = fish fed D15 diet @ 32 ppt salinity; IBW = initial body weight; FBW = final body weight; WG = weight gain; ADG = average daily gain; SGR = specific growth rate; RGR = Relative growth rate; CF = condition factor.

**Table 5:** Feed utilization parameters of Florida red tilapia (♂ and ♀) fed Moringa oleifera leaf ethanol extract (MOLE) for 60 days pre-spawning and 120 days spawning period, at different salinities.

Variable	Salinity	(18ppt)				(32ppt)				*P*-value		
	Diet	D0	D5	D10	D15	D0	D5	D10	D15	Salinity	MOLE	S×M^1^
	Treatment	MSD0	MSD5	MSD10	MSD15	HSD0	HSD5	HSD10	HSD15			
Female ♀												
FI, g		67.7 ± 1.69^a^	57.6 ± 0.61^bcd^	63.2 ± 4.52^ab^	55.4 ± 1.68^cd^	64.7 ± 4.13^ab^	59.3 ± 1.72^b^	52.8 ± 1.5^d^	56.1 ± 2.26^cd^	0.271	0.006	0.093
FCR, g		1.57 ± 0.02^b^	1.37 ± 0.03^cd^	1.33 ± 0.09^cde^	1.06 ± 0.02^f^	1.98 ± 0.06^a^	1.45 ± 0.03^bc^	1.27 ± 0.02^de^	1.21 ± 0.01^e^	0.006	< 0.001	< 0.001
PER, g		2.07 ± 0.03^e^	2.38 ± 0.05^cd^	2.46 ± 0.16^cd^	3.08 ± 0.07^a^	1.65 ± 0.05^f^	2.26 ± 0.05^de^	2.57 ± 0.05^bc^	2.69 ± 0.03^b^	0.239	< 0.001	< 0.001
PPV, %		52.1 ± 2^c^	42.6 ± 0.85^e^	62.9 ± 4.22^b^	74.3 ± 1.39^a^	27.5 ± 1.45^f^	59.1 ± 1.67^b^	45.1 ± 0.516^de^	49.8 ± 0.28^cd^	< 0.001	< 0.001	< 0.001
EU, %		25.5 ± 0.45^b^	25.8 ± 1.04^b^	27.2 ± 1.41^ab^	30 ± 0.81^a^	27 ± 1.45^ab^	25.9 ± 1.16 ^b^	26.2 ± 0.19^b^	25.5 ± 0.46^b^	0.021	0.300	0.166
Male ♂												
FI, g		88.1 ± 0.07^a^	73.7 ± 1.06^bc^	73.7 ± 5.27^bc^	66.4 ± 1.22^bcd^	65.7 ± 0.38^cd^	64.8 ± 0.83^d^	74.3 ± 1.39^b^	82.8 ± 4.31^a^	< 0.001	0.052	0.003
FCR, g		2.33 ± 0.02^a^	1.27 ± 0.04^d^	1.44 ± 0.09^c^	1.30 ± 0.04^d^	2.33 ± 0.03^a^	1.64 ± 0.05^b^	1.20 ± 0.04^de^	1.08 ± 0.024^e^	< 0.001	< 0.001	< 0.001
PER, g		2.1 ± 0.01^d^	2.58 ± 0.06^b^	2.26 ± 0.19^cd^	2.51 ± 0.14^bc^	1.4 ± 0.04^e^	2.0 ± 0.11^d^	2.76 ± 0.06^b^	3.06 ± 0.05^a^	< 0.001	< 0.001	0.766
PPV, %		68.7 ± 0.49^a^	46.2 ± 0.71^ac^	53.1 ± 3.67^b^	57.6 ± 1.68^b^	32.4 ± 2.97^d^	46.2 ± 1.74^c^	59.1 ± 0.75^b^	68.0 ± 2.75^a^	< 0.001	< 0.001	< 0.001
EU, %		40.3 ± 0.54^a^	32.6 ± 0.59^bc^	30.3 ± 2.03^cd^	31.8 ± 1.31^c^	19.2 ± 1.67^e^	27.7 ± 1.27^d^	33.3 ± 0.42^bc^	35.9 ± 1.41^b^	< 0.001	0.017	< 0.001

Means in the same row without a common superscript letter differ significantly (*P <* 0.05) as analyzed by two-way ANOVA and the TUKEY test. MOLE = Moringa oleifera leaf ethanol extract; S × M = Salinity × MOLE interaction effect; D0 = basal diet without MOLE-supplementation (control); D5 = MOLE-supplemented diet with 5 g/kg; D10 = MOLE-supplemented diet with 10 g/kg; D15 = MOLE-supplemented diet with 15 g/kg; Treatments: MSD0 = fish fed D0 diet @ 18ppt salinity; MSD5 = fish fed D5 diet @ 18ppt salinity; MSD10 = fish fed D10 diet @ 18ppt salinity; MSD15 = fish fed D15 diet @ 18ppt salinity; HSD0 = fish fed D0 diet @ 32 ppt salinity; HSD5 = fish fed D5 diet @ 32 ppt salinity; HSD10 = fish fed D10 diet @ 32 ppt salinity; HSD15 = fish fed D15 diet @ 32 ppt salinity; FI = Feed intake; FCR = feed conversion ratio; PER = protein efficiency ratio; PPV = protein productive value; EU = Energy utilization.

**Table 6:** Body chemical composition of Florida red tilapia (♂ and ♀) fed Moringa oleifera leaf ethanol extract (MOLE) for 60 days pre-spawning and 120 days spawning period, at different salinities.

Variable	Salinity	(18ppt)				(32ppt)				*P*-value		
	Diet	D0	D5	D10	D15	D0	D5	D10	D15	Salinity	MOLE	S×M^1^
	Treatment	MSD0	MSD5	MSD10	MSD15	HSD0	HSD5	HSD10	HSD15			
Female ♀												
Moisture		71.7 ± 0.12^b^	71.9 ± 0.06^b^	72.1 ± 0.04^b^	71.7 ± 0.15^b^	68.6 ± 0.33^c^	72.6 ± 0.08^a^	72.2 ± 0.1^ab^	72 ± 0.1^b^	< 0.001	< 0.001	< 0.001
Dry matter, %		28.3 ± 0.12^b^	28.1 ± 0.06^b^	27.9 ± 0.04^b^	28.3 ± 0.15^b^	31.4 ± 0.33^a^	27.4 ± 0.08^c^	27.8 ± 0.1^bc^	28 ± 0.1^b^	< 0.001	< 0.001	< 0.001
Protein, %		57.8 ± 0.22^d^	60.4 ± 0.32^b^	60.8 ± 0.21^ab^	60.8 ± 0.28^ab^	59.2 ± 0.27^c^	61.1 ± 0.1^a^	60.3 ± 0.27^b^	60.8 ± 0.41^ab^	< 0.001	< 0.001	< 0.001
Lipid, %		21 ± 0.35^a^	20.1 ± 0.16^b^	20.2 ± 0.07^b^	19.9 ± 0.04^c^	21.2 ± 0.04^a^	20.0 ± 0.17^b^	20.1 ± 0.06^b^	19 ± 0.13^d^	< 0.001	< 0.001	0.012
Ash, %		14.9 ± 0.44^f^	16.2 ± 0.23^e^	17.9 ± 0.07^c^	18.7 ± 0.12^b^	17.6 ± 0.17^d^	18.1 ± 0.1^c^	19.1 ± 0.23^ab^	19.5 ± 0.21^a^	< 0.001	< 0.001	0.003
Male ♂												
Moisture		70 ± 0.16^f^	72.1 ± 0.15^d^	72.3 ± 0.13^cd^	73.1 ± 0.02^a^	71.6 ± 0.22^e^	72.6 ± 0.13^de^	72.9 ± 0.04^ab^	73.2 ± 0.03^a^	< 0.001	< 0.001	< 0.001
Dry matter, %		30 ± 0.16^a^	27.9 ± 0.15^c^	27.7 ± 0.13^cd^	26.9 ± 0.02^f^	28.4 ± 0.22^b^	27.4 ± 0.13^de^	27.1 ± 0.05^ef^	26.8 ± 0.03^f^	< 0.001	< 0.001	< 0.001
Protein, %		63.3 ± 0.11^d^	64.6 ± 0.35^b^	64.8 ± 0.35^b^	65.7 ± 0.17^a^	64.2 ± 0.352^c^	65.1 ± 0.35^ab^	65.6 ± 0.17^a^	65.8 ± 0.16^a^	0.012	< 0.001	0.451
Lipid, %		18.2 ± 0.15^a^	15.8 ± 0.47^bc^	15.5 ± 0.49^bc^	14.1 ± 0.29^d^	16.4 ± 0.44^b^	15.1 ± 0.51^c^	14.4 ± 0.26^cd^	13.9 ± 0.36^e^	0.004	< 0.001	0.274
Ash, %		18.2 ± 0.1^e^	19.3 ± 0.06^cd^	19.4 ± 0.08^c^	20.1 ± 0.08^ab^	19.1 ± 0.03^d^	19.5 ± 0.1^c^	20 ± 0.15^b^	20.2 ± 0.02^a^	< 0.001	< 0.001	0.003

Means in the same row without a common superscript letter differ significantly (*P <* 0.05) as analyzed by two-way ANOVA and the TUKEY test. MOLE = Moringa oleifera leaf ethanol extract; S × M = Salinity × MOLE interaction effect; D0 = basal diet without MOLE-supplementation (control); D5 = MOLE-supplemented diet with 5 g/kg; D10 = MOLE-supplemented diet with 10 g/kg; D15 = MOLE-supplemented diet with 15 g/kg; Treatments: MSD0 = fish fed D0 diet @ 18ppt salinity; MSD5 = fish fed D5 diet @ 18ppt salinity; MSD10 = fish fed D10 diet @ 18ppt salinity; MSD15 = fish fed D15 diet @ 18ppt salinity; HSD0 = fish fed D0 diet @ 32 ppt salinity; HSD5 = fish fed D5 diet @ 32 ppt salinity; HSD10 = fish fed D10 diet @ 32 ppt salinity; HSD15 = fish fed D15 diet @ 32 ppt salinity.

**Table 7:** Biometric indices of Florida red tilapia (♂ and ♀) fed Moringa oleifera leaf ethanol extract (MOLE) for 60 days pre-spawning and 120 days spawning period, at different salinities.

Variable		Salinity	(18ppt)				(32ppt)				*P*-value		
		Diet	D0	D5	D10	D15	D0	D5	D10	D15	Salinity	MOLE	S×M^1^
		Treatment	MSD0	MSD5	MSD10	MSD15	HSD0	HSD5	HSD10	HSD15			
Female ♀													
HSI, %			2.67 ± 0.01^b^	2.49 ± 0.02^d^	2.38 ± 0.04^e^	2.17 ± 0.03^f^	2.81 ± 0.02^a^	2.72 ± 0.00^b^	2.65 ± 0.03^b^	2.57 ± 0.02^c^	< 0.001	< 0.001	< 0.001
VSI, %			7.59 ± 0.13^b^	7.02 ± 0.03^cd^	6.65 ± 0.04^e^	6.32 ± 0.05^f^	8.19 ± 0.12^a^	7.79 ± 0.13^b^	7.1 ± 0.06^c^	6.74 ± 0.14^de^	< 0.001	< 0.001	0.293
GSI, %			4.85 ± 0.04^c^	5.94 ± 0.1^a^	5.88 ± 0.08^a^	5.9 ± 0.04^a^	3.95 ± 0.10^d^	4.93 ± 0.1^c^	5.29 ± 0.16^b^	5.74 ± 0.09^a^	< 0.001	< 0.001	0.002
Male ♂													
HSI, %			1.64 ± 0.01^cd^	1.59 ± 0.012^de^	1.51 ± 0.01^f^	1.44 ± 0.01^g^	1.82 ± 0.02^a^	1.7 ± 0.0145^b^	1.66 ± 0.03^bc^	1.58 ± 0.012^e^	< 0.001	< 0.001	0.181
VSI, %			6.36 ± 0.01^b^	6.06 ± 0.04^c^	5.61 ± 0.03^d^	5.27 ± 0.09^e^	6.9 ± 0.02^a^	6.54 ± 0.01^b^	6.43 ± 0.04^b^	6.16 ± 0.115^c^	< 0.001	< 0.001	0.005
TSI, %			1.1 ± 0.02^e^	2.02 ± 0.05^b^	2.07 ± 0.06^b^	2.26 ± 0.04^a^	0.87 ± 0.03^f^	1.44 ± 0.06^d^	1.65 ± 0.02^c^	2.06 ± 0.07^b^	< 0.001	< 0.001	0.003

Means in the same row without a common superscript letter differ significantly (*P <* 0.05) as analyzed by two-way ANOVA and the TUKEY test. MOLE = Moringa oleifera leaf ethanol extract; S × M = Salinity × MOLE interaction effect; D0 = basal diet without MOLE-supplementation (control); D5 = MOLE-supplemented diet with 5 g/kg; D10 = MOLE-supplemented diet with 10 g/kg; D15 = MOLE-supplemented diet with 15 g/kg; Treatments: MSD0 = fish fed D0 diet @ 18ppt salinity; MSD5 = fish fed D5 diet @ 18ppt salinity; MSD10 = fish fed D10 diet @ 18ppt salinity; MSD15 = fish fed D15 diet @ 18ppt salinity; HSD0 = fish fed D0 diet @ 32 ppt salinity; HSD5 = fish fed D5 diet @ 32 ppt salinity; HSD10 = fish fed D10 diet @ 32 ppt salinity; HSD15 = fish fed D15 diet @ 32 ppt salinity; HSI = Hepatosomatic Index; VSI = Viscerosomatic Index; TSI = Testssomatic Index; GSI = Gonadosomatic Index.

**Table 8:** Hematobiochemical (complete blood count) analysis of Florida red tilapia (♂ and ♀) fed Moringa oleifera leaf ethanol extract (MOLE) for 60 days pre-spawning and 120 days spawning period, at different salinities.

Variable	Salinity	(18ppt)				(32ppt)				*P*-value		
	Diet	D0	D5	D10	D15	D0	D5	D10	D15	Salinity	MOLE	S×M^1^
	Treatment	MSD0	MSD5	MSD10	MSD15	HSD0	HSD5	HSD10	HSD15			
Female ♀												
RBCs (10^6^mm^−3^)		2.74 ± 0.01^c^	2.93 ± 0.03^b^	3.07 ± 0.05^a^	3.13 ± 0.01^a^	1.91 ± 0.01^f^	2.42 ± 0.05^e^	2.6 ± 0.03^d^	2.74 ± 0.01^c^	< 0.001	< 0.001	< 0.001
Hb (g dL ^1^)		7.89 ± 0.12^c^	8.29 ± 0.2^c^	9.05 ± 0.47^b^	9.79 ± 0.06^a^	6.31 ± 0.06^e^	6.99 ± 0.07^d^	8.26 ± 0.07^c^	9.09 ± 0.04^b^	< 0.001	< 0.001	0.108
Hct (%)		28.5 ± 0.07^b^	29 ± 0.09^b^	29.4 ± 0.21^a^	29.8 ± 0.07^a^	18.1 ± 0.06^e^	21 ± 0.19^d^	21.6 ± 0.29^c^	28.5 ± 0.07^b^	< 0.001	< 0.001	< 0.001
WBCs (10^3^mm^−3^)		34.5 ± 0.29^b^	35.9 ± 0.08^a^	36.4 ± 0.17^a^	36.8 ± 0.01^a^	30.2 ± 0.58^c^	34 ± 0.32^b^	34 ± 0.27^b^	34.5 ± 0.50^b^	< 0.001	< 0.001	0.009
Lymphocyte (%)		49.7 ± 0.22^c^	51.9 ± 0.92^b^	53.7 ± 0.13^a^	54.7 ± 0.35^a^	45.1 ± 0.59^e^	48.7 ± 0.32^cd^	48 ± 0.46^d^	51.3 ± 0.24^b^	< 0.001	< 0.001	0.065
Monocyte (%)		7.44 ± 0.34^bc^	8.07 ± 0.04^ab^	8.36 ± 0.03^a^	8.47 ± 0.06^a^	5.33 ± 0.33^d^	6.67 ± 0.33^c^	6.91 ± 0.22^c^	7.07 ± 0.26^c^	< 0.001	< 0.001	0.399
Neutrophil (%)		40.7 ± 0.33^c^	38 ± 0.07^d^	36.8 ± 0.26^e^	35.8 ± 0.30^e^	47.9 ± 0.66^a^	42.9 ± 0.3^b^	43.4 ± 0.26^b^	39.9 ± 0.31^c^	< 0.001	< 0.001	0.001
MCV		104 ± 0.28^a^	98.9 ± 0.973^b^	96.1 ± 1.26^bc^	95.2 ± 0.33^c^	94.6 ± 0.39^c^	86.7 ± 2.65^d^	83.1 ± 0.13^e^	104 ± 0.28^a^	< 0.001	< 0.001	< 0.001
MCH		28.7 ± 0.42^c^	28.3 ± 0.88^c^	29.5 ± 1.1^bc^	31.2 ± 0.16^ab^	33 ± 0.33^a^	28.9 ± 0.58^c^	31.7 ± 0.64^a^	33.1 ± 0.01^a^	< 0.001	< 0.001	0.06
MCHC		27.7 ± 0.42^e^	28.6 ± 0.63^e^	30.7 ± 1.49^d^	32.8 ± 0.12^bcd^	34.8 ± 0.29^b^	33.3 ± 0.49^bc^	38.2 ± 0.83^a^	31.9 ± 0.08^cd^	< 0.001	< 0.001	< 0.001
Male ♂												
RBCs (10^6^mm^−3^)		2.88 ± 0.02^c^	3.07 ± 0.04^b^	3.34 ± 0.02^b^	3.35 ± 0.08^a^	1.93 ± 0.01^e^	2.36 ± 0.06^d^	2.85 ± 0.04^c^	2.9 ± 0.06^c^	< 0.001	< 0.001	< 0.001
Hb (g dL ^1^)		9.09 ± 0.04^cd^	9.19 ± 0.04^c^	9.53 ± 0.04^b^	9.73 ± 0.06^a^	6.12 ± 0.04^f^	7.46 ± 0.00^e^	7.68 ± 0.09^e^	9 ± 0.09^d^	< 0.001	< 0.001	< 0.001
Hct (%)		23.4 ± 0.41^d^	25.2 ± 0.58^cd^	26.5 ± 0.71^abc^	28.2 ± 1^a^	17.4 ± 0.25^f^	20.7 ± 0.24^e^	26.1 ± 0.59^bc^	28.1 ± 0.94^ab^	< 0.001	< 0.001	< 0.001
WBCs (10^3^mm^−3^)		27.6 ± 0.84^d^	29.3 ± 0.15^c^	30.1 ± 0.06^bc^	33.5 ± 0.3^a^	21.3 ± 0.15^f^	24 ± 0.25^e^	28.8 ± 0.8^cd^	31.5 ± 0.8^b^	< 0.001	< 0.001	< 0.001
Lymphocyte (%)		66.4 ± 0.76^c^	67.6 ± 0.14^bc^	70 ± 0.109^ab^	71.7 ± 0.25^a^	56.6 ± 2.08^e^	60.7 ± 0.46^d^	67.7 ± 0.33^bc^	70.3 ± 0.48^ab^	< 0.001	< 0.001	< 0.001
Monocyte (%)		3.56 ± 0.08^b^	3.7 ± 0.04^ab^	3.68 ± 0.08^ab^	3.87 ± 0.03^a^	2.65 ± 0.17^d^	3.26 ± 0.02^c^	3.49 ± 0.02^b^	3.59 ± 0.03^b^	< 0.001	< 0.001	0.001
Neutrophil (%)		27.9 ± 0.6^c^	26.3 ± 0.32^cd^	23.7 ± 0.36^e^	22.3 ± 0.46^e^	38.6 ± 1.86^a^	33.6 ± 0.31^b^	26.2 ± 0.11^cd^	24 ± 0.41^de^	< 0.001	< 0.001	< 0.001
MCV		78.6 ± 1.79^d^	81.9 ± 2.18^cd^	79.3 ± 1.87^d^	84.2 ± 4.85^bcd^	90.1 ± 1.84^abc^	88 ± 1.16^abcd^	91.8 ± 3.35^ab^	96.9 ± 4.5^a^	< 0.001	0.18	0.666
MCH		30.5 ± 0.33^ab^	29.9 ± 0.47^ab^	28.6 ± 0.19^bc^	29.1 ± 0.86^b^	31.7 ± 0.30^a^	31.6 ± 0.78^a^	27 ± 0.66^c^	31.1 ± 0.87^a^	0.065	< 0.001	0.036
MCHC		38.8 ± 0.51^a^	36.6 ± 0.72^b^	36.1 ± 0.84^b^	34.6 ± 1.03^b^	35.2 ± 0.47^b^	36 ± 0.42^b^	29.5 ± 0.38^d^	32.1 ± 0.8^c^	< 0.001	< 0.001	0.004

Means in the same row without a common superscript letter differ significantly (*P <* 0.05) as analyzed by two-way ANOVA and the TUKEY test. MOLE = Moringa oleifera leaf ethanol extract; S × M = Salinity × MOLE interaction effect; D0 = basal diet without MOLE-supplementation (control); D5 = MOLE-supplemented diet with 5 g/kg; D10 = MOLE-supplemented diet with 10 g/kg; D15 = MOLE-supplemented diet with 15 g/kg; Treatments: MSD0 = fish fed D0 diet @ 18ppt salinity; MSD5 = fish fed D5 diet @ 18ppt salinity; MSD10 = fish fed D10 diet @ 18ppt salinity; MSD15 = fish fed D15 diet @ 18ppt salinity; HSD0 = fish fed D0 diet @ 32 ppt salinity; HSD5 = fish fed D5 diet @ 32 ppt salinity; HSD10 = fish fed D10 diet @ 32 ppt salinity; HSD15 = fish fed D15 diet @ 32 ppt salinity.

**Table 9:** Total cholesterol, liver enzymes (ALT and AST) and kidney function (Urea and uric acid) indices and stress marker (cortisol) of Florida red tilapia (♂ and ♀) fed Moringa oleifera leaf ethanol extract (MOLE) for 60 days pre-spawning and 120 days spawning period, at different salinities.

Variable	Salinity	(18ppt)				(32ppt)				*P*-value		
	Diet	D0	D5	D10	D15	D0	D5	D10	D15	Salinity	MOLE	S×M^1^
	Treatment	MSD0	MSD5	MSD10	MSD15	HSD0	HSD5	HSD10	HSD15			
Female ♀												
Cholesterol mg/dl		83 ± 0.58^c^	80 ± 0.58^d^	78.7 ± 0.88^d^	74 ± 1.53^e^	135 ± 1.2^a^	110 ± 1.45^b^	81.7 ± 0.88^cd^	85.3 ± 2.03^c^	< 0.001	< 0.001	< 0.001
AST (IU/L)		62.7 ± 0.56^b^	58 ± 0.58^c^	56 ± 0.58^cd^	53.7 ± 0.88^d^	88.3 ± 0.64^a^	61.7 ± 0.02^b^	60.5 ± 0.25^b^	59.9 ± 0.38^b^	< 0.001	< 0.001	< 0.001
ALT (IU/L)		38.9 ± 0.21^b^	31.3 ± 0.33^cd^	31 ± 0.58^cd^	27.3 ± 0.33^d^	59.4 ± 0.56^a^	33.9 ± 0.8^c^	31.5 ± 0.82^cd^	30.7 ± 0.83^cd^	< 0.001	< 0.001	< 0.001
Urea (mg/dl)		40.5 ± 0.29^b^	36.6 ± 0.33^c^	34.6 ± 0.28^c^	33.8 ± 0.34^c^	46.8 ± 3.03^a^	45.2 ± 1.3^a^	40.5 ± 1.73^b^	40.5 ± 0.29^b^	< 0.001	< 0.001	0.748
Uric Acid mg/dl		2.56 ± 0.0^b^	2.14 ± 0.05^bc^	1.76 ± 0.08^c^	1.68 ± 0.07^c^	3.82 ± 0.36^a^	3.58 ± 0.16^a^	2.65 ± 0.32^b^	2.56 ± 0.0^b^	< 0.001	< 0.001	0.356
Cortisol (pg mL^−1^)		213 ± 1.44^d^	196 ± 4.58^e^	184 ± 10.1^e^	171 ± 1.2^f^	282 ± 1.88^a^	258 ± 1.73^b^	235 ± 0.43^c^	213 ± 1.44^d^	< 0.001	< 0.001	0.025
Male ♂												
Cholesterol mg/dl		112 ± 0.58^c^	106 ± 3.18^d^	91.3 ± 1.86^e^	91.7 ± 0.88^e^	144 ± 1.2^a^	116 ± 0.33^bc^	116 ± 1.2^bc^	118 ± 2.03^b^	< 0.001	< 0.001	< 0.001
AST (IU/L)		65 ± 0.46^b^	59.7 ± 0.33^c^	57.3 ± 1.2^c^	53 ± 2.52^d^	88.4 ± 0.59^a^	63.4 ± 0.24^b^	59.5 ± 0.51^c^	58.6 ± 0.65^c^	< 0.001	< 0.001	< 0.001
ALT (IU/L)		33.9 ± 0.32^b^	31.3 ± 0.33^c^	31 ± 0.58^c^	30.3 ± 0.33^c^	56.6 ± 1.21^a^	33.5 ± 0.25^b^	31.5 ± 0.48^c^	30.2 ± 0.49^c^	< 0.001	< 0.001	< 0.001
Urea (mg/dl)		40.1 ± 1.09^c^	38.5 ± 0.54^cd^	36.9 ± 0.23^d^	36.1 ± 0.01^d^	45 ± 0.66^a^	42.8 ± 0.15^b^	40.4 ± 0.72^c^	39.3 ± 0.4^c^	< 0.001	< 0.001	0.463
Uric Acid (mg/dl)		2.79 ± 0.09^c^	2.42 ± 0.06^d^	2.2 ± 0.07^e^	2.04 ± 0.04^e^	3.54 ± 0.07^a^	3.22 ± 0.03^b^	2.8 ± 0.04^c^	2.66 ± 0.04^c^	< 0.001	< 0.001	0.28
Cortisol (pg mL ^1^)		218 ± 1.05^b^	150 ± 29.6^c^	204 ± 2.96^b^	192 ± 3.51^b^	286 ± 2.16^a^	258 ± 3.48^a^	215 ± 0.66^b^	209 ± 1.23^b^	< 0.001	0.001	0.001

Means in the same row without a common superscript letter differ significantly (*P <* 0.05) as analyzed by two-way ANOVA and the TUKEY test. MOLE = Moringa oleifera leaf ethanol extract; S × M = Salinity × MOLE interaction effect; D0 = basal diet without MOLE-supplementation (control); D5 = MOLE-supplemented diet with 5 g/kg; D10 = MOLE-supplemented diet with 10 g/kg; D15 = MOLE-supplemented diet with 15 g/kg; Treatments: MSD0 = fish fed D0 diet @ 18ppt salinity; MSD5 = fish fed D5 diet @ 18ppt salinity; MSD10 = fish fed D10 diet @ 18ppt salinity; MSD15 = fish fed D15 diet @ 18ppt salinity; HSD0 = fish fed D0 diet @ 32 ppt salinity; HSD5 = fish fed D5 diet @ 32 ppt salinity; HSD10 = fish fed D10 diet @ 32 ppt salinity; HSD15 = fish fed D15 diet @ 32 ppt salinity; ALT = glutamic-pyruvic transaminase; AST = aspartate aminotransferase.

**Table 10:** Reproductive performance (fecundity, spawning, Inter-spawning-interval, days to hatch, hatchability, yolk-sac absorption days, and larval length) of female (♀) Floria red tilapia fed moringa oleifera leaf ethanol extract (MOLE) for 60 days at different salinities during the pre-spawning period and continued spawning season for 120 days.

Variable	Salinity Diet Treatment	(18ppt)				(32ppt)				*P*-value		
		D0	D5	D10	D15	D0	D5	D10	D15	Salinity	MOLE	S×M^1^
		MSD0	MSD5	MSD10	MSD15	HSD0	HSD5	HSD10	HSD15			
Time to first spawning (day)		31.3 ± 0.88^d^	24 ± 0.58^e^	23.7 ± 0.88^e^	21 ± 0.577^f^	57 ± 0.58^a^	44 ± 0.58^b^	36.7 ± 0.88^c^	34.7 ± 0.88^c^	< 0.001	< 0.001	< 0.001
Inter-spawning intervals (days)		18 ± 0^b^	16 ± 0^cd^	14.3 ± 0.33^de^	13 ± 0.58^e^	27 ± 1.15^a^	17 ± 0.58^bc^	17 ± 0.58^bc^	15.3 ± 0.33^cd^	< 0.001	< 0.001	< 0.001
Number of spawning ♀/group		30 ± 2.89^c^	40 ± 2^b^	49 ± 0^a^	53.3 ± 2.67^a^	10 ± 1.15^d^	28.3 ± 4.41^c^	35 ± 0^bc^	38 ± 2^b^	< 0.001	< 0.001	0.372
Average spawn times per 1♀		3.33 ± 0.32^c^	4.45 ± 0.22^b^	5.44 ± 0^a^	5.63 ± 0.297^a^	1.11 ± 0.13^d^	3.15 ± 0.49^c^	3.89 ± 0^bc^	4.19 ± 0.243^b^	< 0.001	< 0.001	0.34
Total number of egg/1 ♀		2350 ± 55.5^de^	2590 ± 41.3^cd^	3320 ± 151^b^	3850 ± 126^a^	688 ± 121^f^	2180 ± 131^e^	2480 ± 10^de^	2830 ± 140^c^	< 0.001	< 0.001	< 0.001
Total number of eggs/group (all ♀)		14,056 ± 1030^b^	17,262 ± 849^b^	23,240 ± 1060^a^	27,069 ± 3070^a^	2947 ± 434^c^	13,226 ± 2010^b^	17,360 ± 70^b^	17,963 ± 1540^b^	< 0.001	< 0.001	0.137
Average number of eggs per 1spawn ♀		471 ± 11.1^ab^	432 ± 6.89^b^	474 ± 21.6^ab^	505 ± 40.1^a^	292 ± 9.46^c^	468 ± 10.4^ab^	496 ± 2^ab^	472 ± 23.4^ab^	0.013	< 0.001	< 0.001
Average absolute fecundity		1562 ± 114^b^	1918 ± 94.3^b^	2582 ± 118^a^	3008 ± 341^a^	327 ± 48.2^c^	1469 ± 223^b^	1929 ± 7.78^b^	1995 ± 172^b^	< 0.001	< 0.001	0.137
Relative fecundity		9.00 ± 0.655^bc^	11.3 ± 0.521^b^	15.0 ± 0.694^a^	17.3 ± 1.99^a^	1.33 ± 0.212^d^	7.00 ± 0.992^c^	10.00 ± 0.0517^bc^	10.67 ± 0.894^b^	< 0.001	< 0.001	0.261
Total number of fry/group (all spawn ♀)		11,785 ± 724^bc^	14,843 ± 701^b^	20,207 ± 789^a^	23,418 ± 2460^a^	1894 ± 279^d^	10,118 ± 1620^c^	14,062 ± 152^b^	14,652 ± 1150^b^	< 0.001	< 0.001	0.169
Average final body weigh per ♀		168 ± 0.98^e^	169 ± 0.57^e^	171 ± 0.363^e^	171 ± 0.35^e^	229 ± 5.82^a^	205 ± 2.84^b^	194 ± 0.64^c^	182 ± 2.04^d^	< 0.001	< 0.001	< 0.001
Average days to hatch/group		6.33 ± 0.33^b^	6.33 ± 0.33^b^	6.67 ± 0.33^ab^	7.67 ± 0.33^a^	4.33 ± 0.33^c^	5.67 ± 0.33^b^	6.33 ± 0.33^b^	6.33 ± 0.67^b^	0.001	0.004	0.19
Hatchability (%)/group		84 ± 1.53^ab^	86 ± 0.58^a^	87 ± 0.58^a^	86.7 ± 0.88^a^	64.3 ± 1.2^e^	76.3 ± 0.88^d^	81 ± 0.58^c^	81.7 ± 0.88^bc^	< 0.001	< 0.001	< 0.001
Yolk-sac absorption (days)		7 ± 0.577^e^	9 ± 0^bc^	10 ± 0^ab^	10.7 ± 0.33^a^	3.67 ± 0.33^f^	7.67 ± 0.33^de^	8.67 ± 0.33^cd^	10 ± 0.58^ab^	< 0.001	< 0.001	0.014
Larval length (mm)		9.66 ± 0.32^c^	10.8 ± 0.09^b^	10.8 ± 0.15^b^	11.9 ± 0.09^a^	7.2 ± 0.18^d^	10.2 ± 0.3^bc^	10.9 ± 0.26^b^	10.8 ± 0.3^b^	< 0.001	< 0.001	< 0.001

Means in the same row without a common superscript letter differ significantly (*P <* 0.05) as analyzed by two-way ANOVA and the TUKEY test. MOLE = Moringa oleifera leaf ethanol extract; S × M = Salinity × MOLE interaction effect; D0 = basal diet without MOLE-supplementation (control); D5 = MOLE-supplemented diet with 5 g/kg; D10 = MOLE-supplemented diet with 10 g/kg; D15 = MOLE-supplemented diet with 15 g/kg; Treatments: MSD0 = fish fed D0 diet @ 18ppt salinity; MSD5 = fish fed D5 diet @ 18ppt salinity; MSD10 = fish fed D10 diet @ 18ppt salinity; MSD15 = fish fed D15 diet @ 18ppt salinity; HSD0 = fish fed D0 diet @ 32 ppt salinity; HSD5 = fish fed D5 diet @ 32 ppt salinity; HSD10 = fish fed D10 diet @ 32 ppt salinity; HSD15 = fish fed D15 diet @ 32 ppt salinity; ISI = Inter-spawning intervals.

**Table 11:** Chemical composition of eggs of female (♀) Florida red tilapia Florida fed moringa oleifera leaf ethanol extract (MOLE) for 60 days pre-spawning and 120 days spawning period, at different salinities.

Variable	Salinity	(18ppt)				(32ppt)						*P* value
	Diet	D0	D5	D10	D15	D0	D5	D10	D15	Salinity	MOLE	S×M
	Treatment	MSD0	MSD5	MSD10	MSD15	HSD0	HSD5	HSD10	HSD15			
Moisture, %		59.7 ± 0.34^bc^	60.4 ± 0.09^a^	60.7 ± 0.04^a^	60.5 ± 0.28^ab^	59.8 ± 0.23^b^	59.8 ± 0.14^bc^	59.9 ± 0.32^abc^	60 ± 0.402^ab^	0.009	0.039	0.798
Dry matter, %		40.3 ± 0.34^a^	39.6 ± 0.09^b^	39.3 ± 0.04^c^	39.5 ± 0.28^b^	40.2 ± 0.23^a^	40.2 ± 0.14^a^	40.1 ± 0.32^ab^	40 ± 0.402^ab^	0.009	0.039	0.798
Protein, %		64.1 ± 0.178^c^	64.6 ± 0.14^b^	65.1 ± 0.06^ab^	65.8 ± 0.36^a^	58.6 ± 0.65^d^	64.6 ± 0.15^b^	65.3 ± 0.05^ab^	65.2 ± 0.15^a^	< 0.001	< 0.001	< 0.001
Lipid, %		32.4 ± 0.21^b^	30.8 ± 0.13^c^	30.2 ± 0.08^cd^	29.5 ± 0.32^d^	34.8 ± 0.21^a^	32.1 ± 0.2^b^	32 ± 0.68^b^	31.9 ± 0.47^b^	< 0.001	< 0.001	0.382
Ash, %		3.15 ± 0.04^d^	3.44 ± 0.02^c^	3.56 ± 0.03^bc^	3.64 ± 0.05^b^	4.26 ± 0.04^a^	3.11 ± 0.06^d^	3.03 ± 0.11^d^	3.06 ± 0.1^d^	0.08	< 0.001	< 0.001

Means in the same row without a common superscript letter differ significantly (*P <* 0.05) as analyzed by two-way ANOVA and the TUKEY test. MOLE = Moringa oleifera leaf ethanol extract; S × M = Salinity × MOLE interaction effect; D0 = basal diet without MOLE-supplementation (control); D5 = MOLE-supplemented diet with 5 g/kg; D10 = MOLE-supplemented diet with 10 g/kg; D15 = MOLE-supplemented diet with 15 g/kg; Treatments: MSD0 = fish fed D0 diet @ 18ppt salinity; MSD5 = fish fed D5 diet @ 18ppt salinity; MSD10 = fish fed D10 diet @ 18ppt salinity; MSD15 = fish fed D15 diet @ 18ppt salinity; HSD0 = fish fed D0 diet @ 32 ppt salinity; HSD5 = fish fed D5 diet @ 32 ppt salinity; HSD10 = fish fed D10 diet @ 32 ppt salinity; HSD15 = fish fed D15 diet @ 32 ppt salinity. 3.64 ± 0.03^d^.

## Data Availability

The data used and/or analyzed in the current study are available from the corresponding author upon reasonable request and data developed during this investigation are comprised in this published paper.
